# Supplementary material for: Ventilation during COVID-19 in a school for students with intellectual and developmental disabilities (IDD)
Source: medRxiv. 2023 Sep 20:2023.09.08.23295268. Originally published 2023 Sep 10. Preprint. [Version 2] doi: 10.1101/2023.09.08.23295268 (PMC10508805; doi:10.1101/2023.09.08.23295268)
Supplement: Supplement 1 [file media-1.pdf]

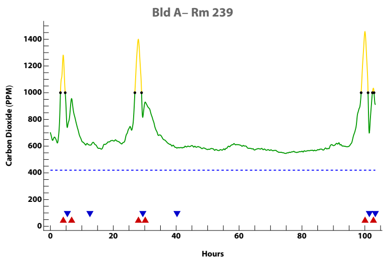

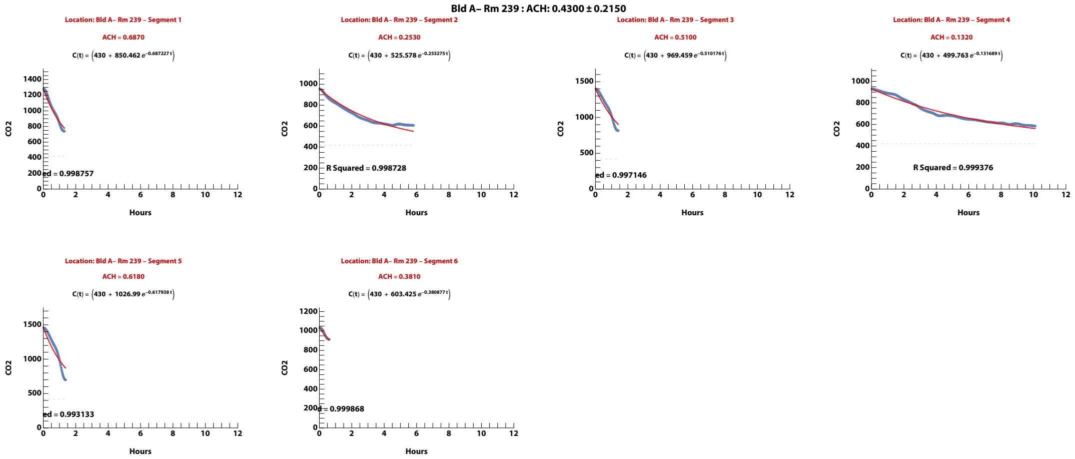

Zand, et al. (2023). Ventilation during COVID–19 in a school for students with intellectual and developmental disabilities (IDD) [Submitted]

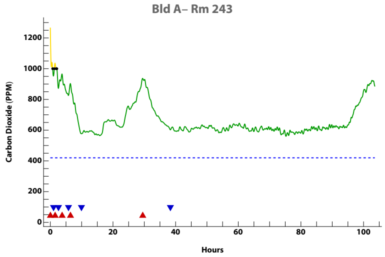

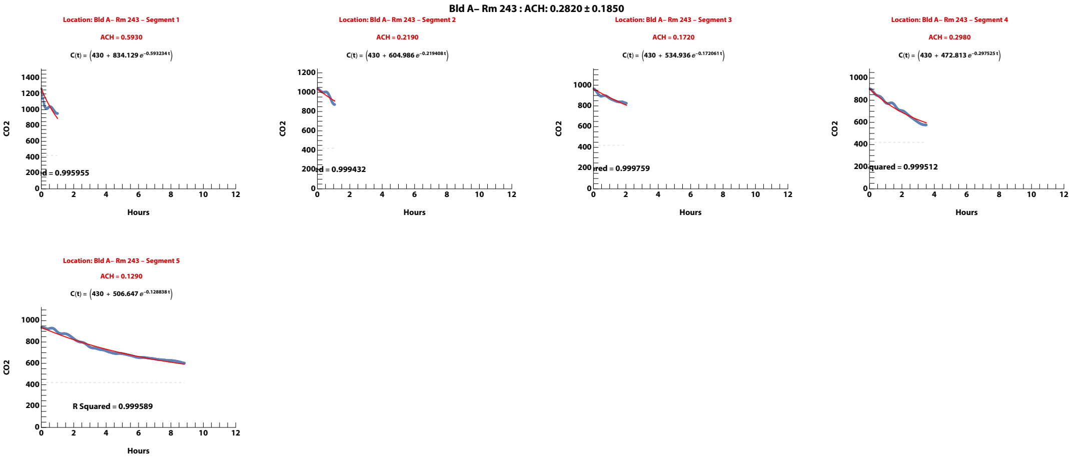

Zand, et al. (2023). Ventilation during COVID–19 in a school for students with intellectual and developmental disabilities (IDD) [Submitted]

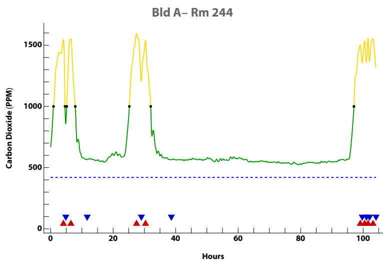

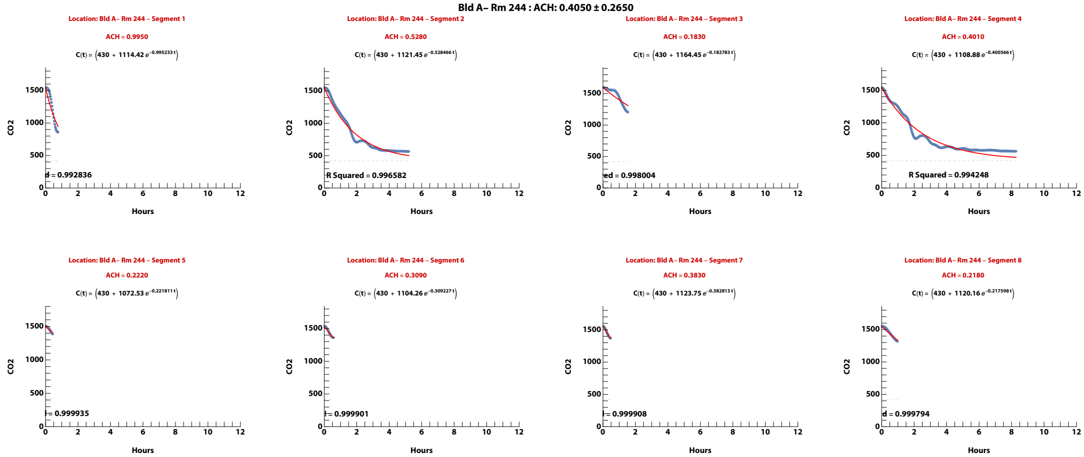

Zand, et al. (2023). Ventilation during COVID–19 in a school for students with intellectual and developmental disabilities (IDD) [Submitted]

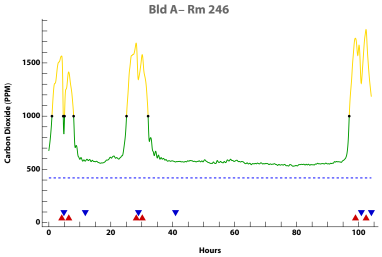

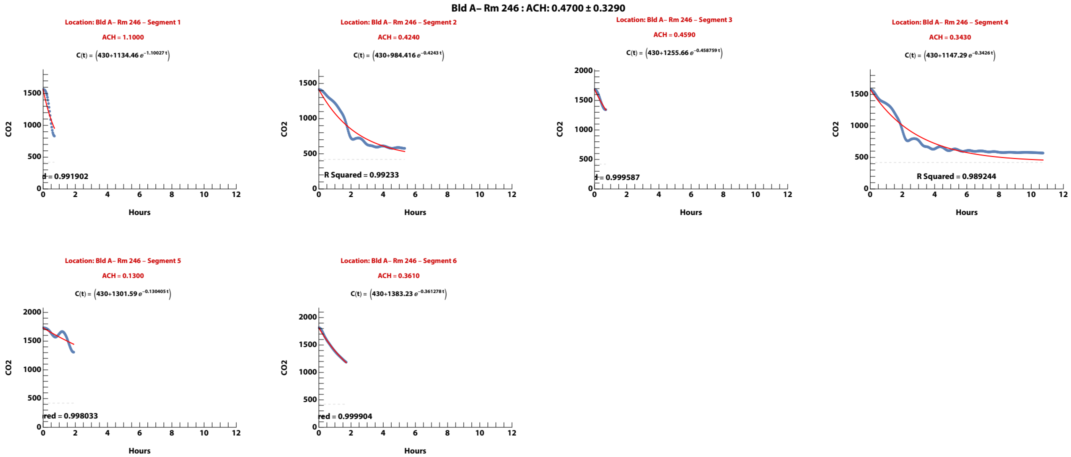

Zand, et al. (2023). Ventilation during COVID–19 in a school for students with intellectual and developmental disabilities (IDD) [Submitted]

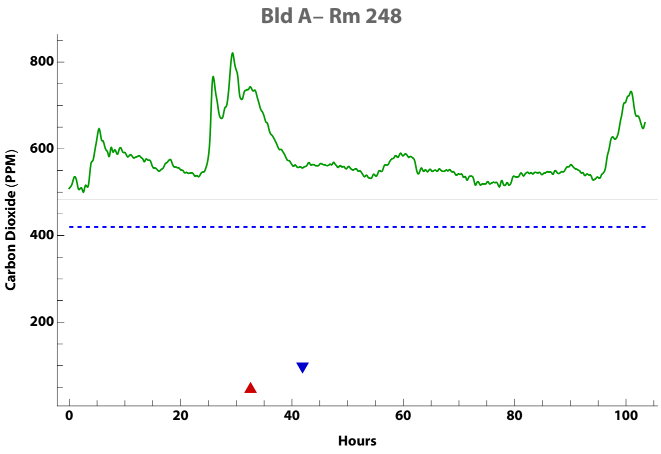

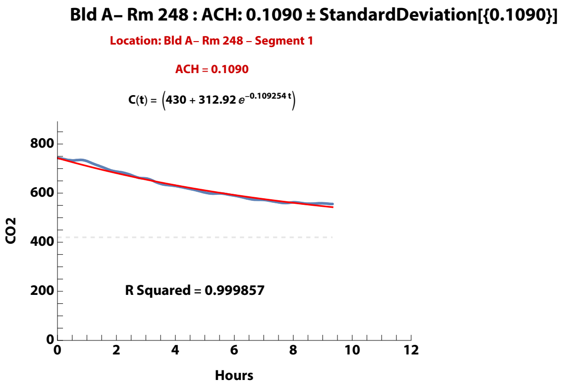

Zand, et al. (2023). Ventilation during COVID–19 in a school for students with intellectual and developmental disabilities (IDD) [Submitted]

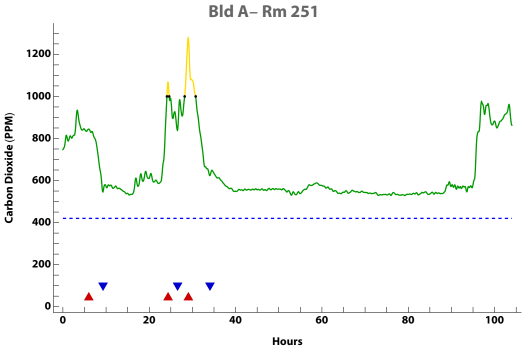

Bld A– Rm 251 : ACH: 0.2290 ± 0.0524

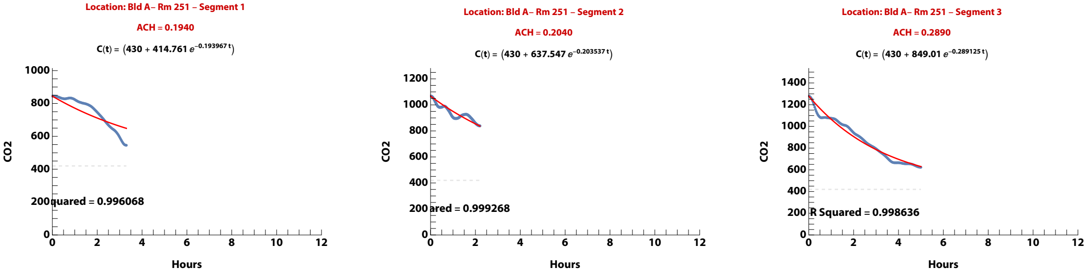

Zand, et al. (2023). Ventilation during COVID–19 in a school for students with intellectual and developmental disabilities (IDD) [Submitted]

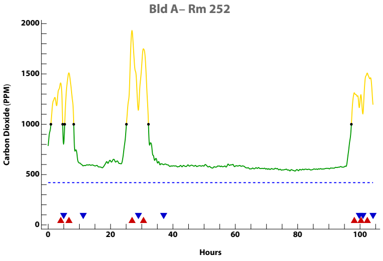

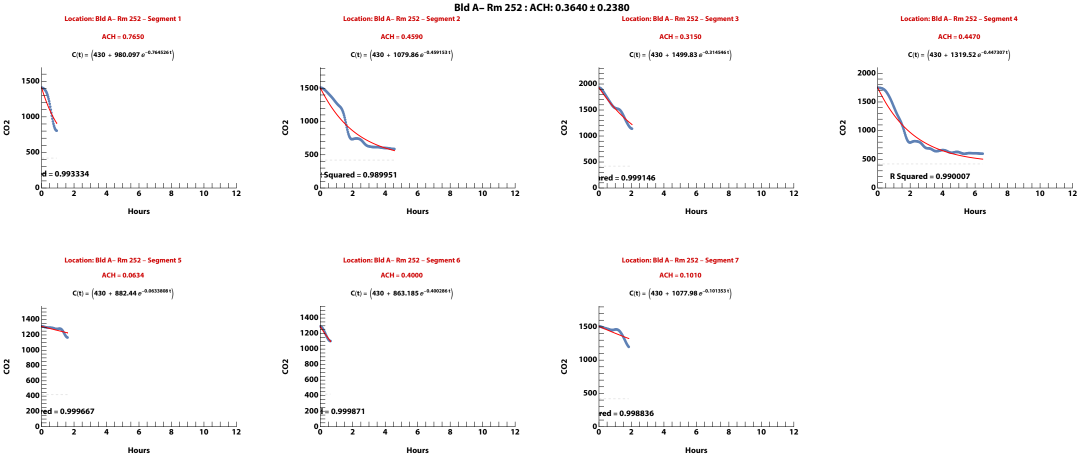

Zand, et al. (2023). Ventilation during COVID–19 in a school for students with intellectual and developmental disabilities (IDD) [Submitted]

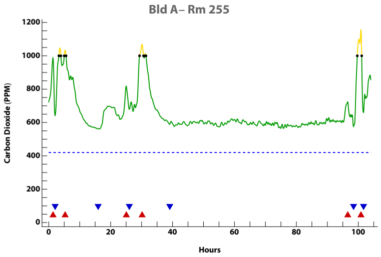

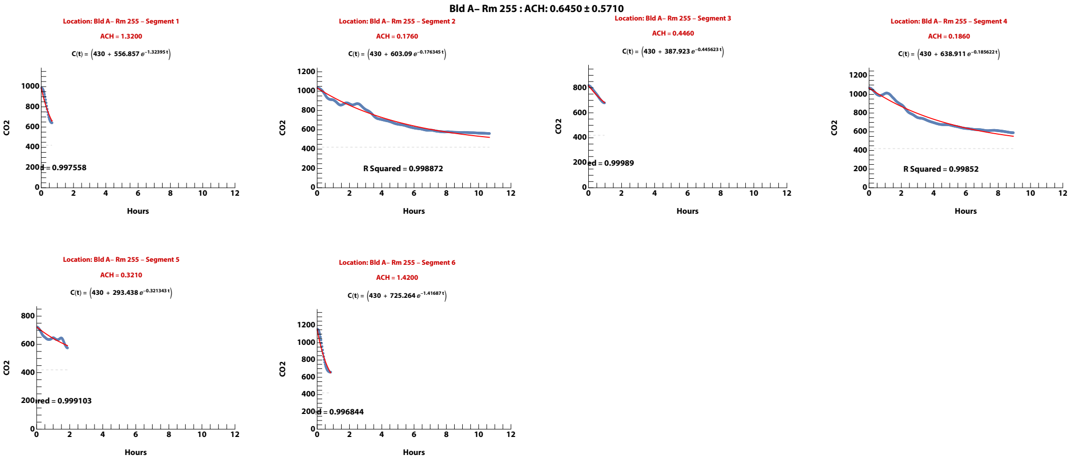

Zand, et al. (2023). Ventilation during COVID–19 in a school for students with intellectual and developmental disabilities (IDD) [Submitted]

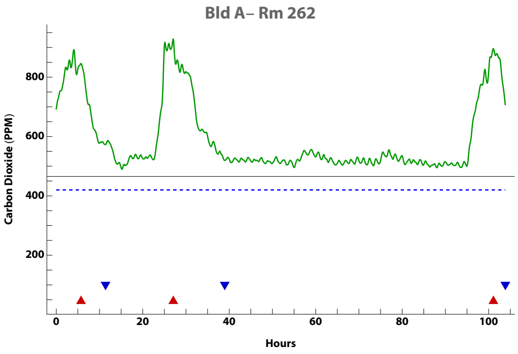

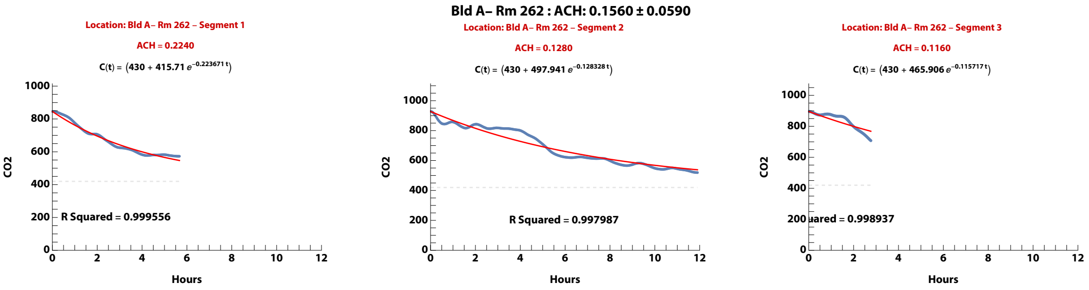

Zand, et al. (2023). Ventilation during COVID–19 in a school for students with intellectual and developmental disabilities (IDD) [Submitted]

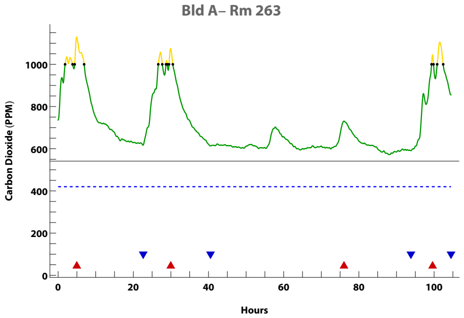

Bld A– Rm 263 : ACH: 0.0947 ± 0.0385

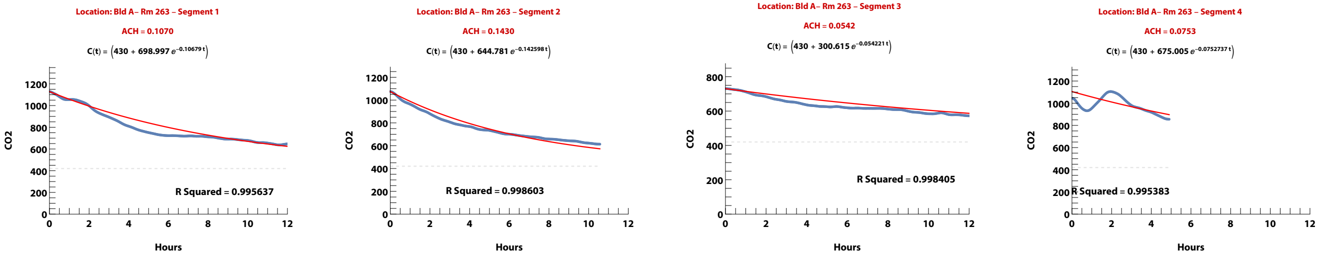

Zand, et al. (2023). Ventilation during COVID–19 in a school for students with intellectual and developmental disabilities (IDD) [Submitted]

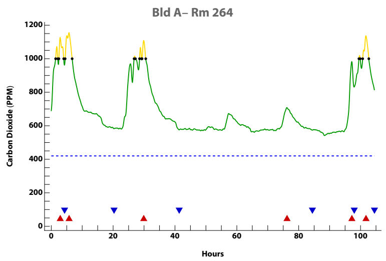

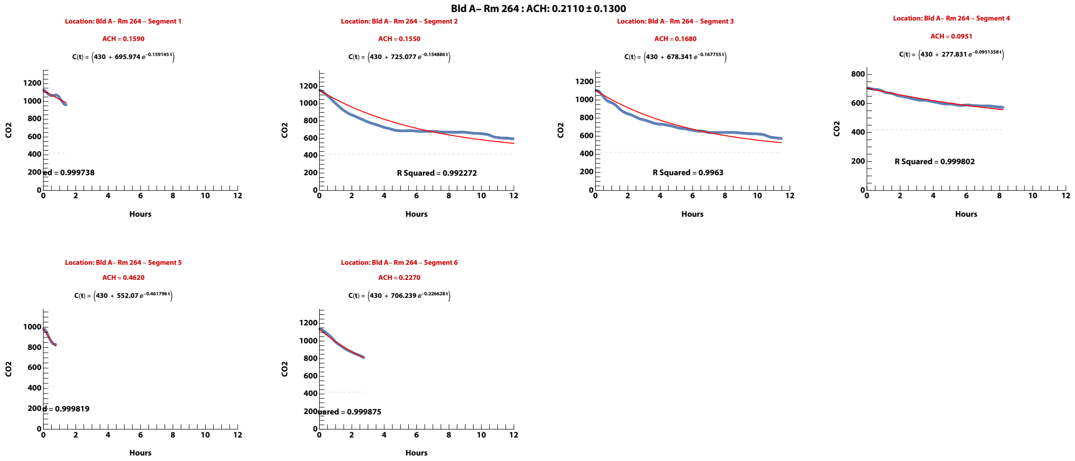

Zand, et al. (2023). Ventilation during COVID–19 in a school for students with intellectual and developmental disabilities (IDD) [Submitted]

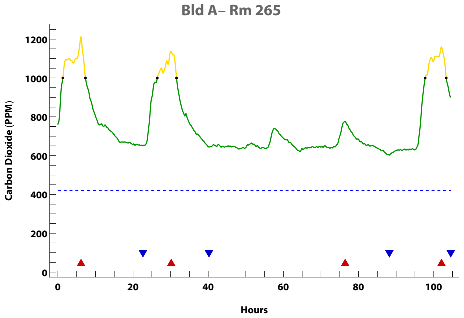

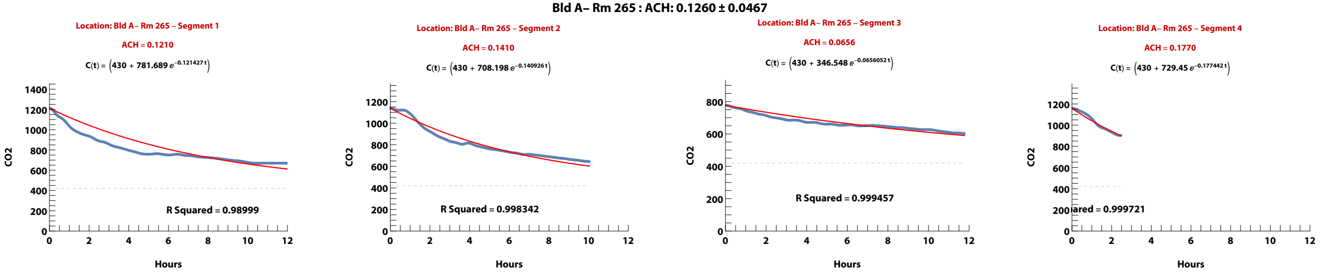

Zand, et al. (2023). Ventilation during COVID–19 in a school for students with intellectual and developmental disabilities (IDD) [Submitted]

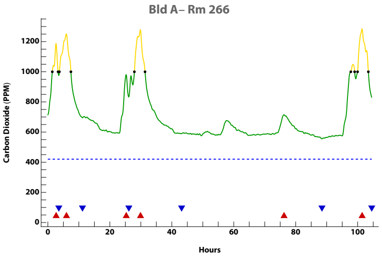

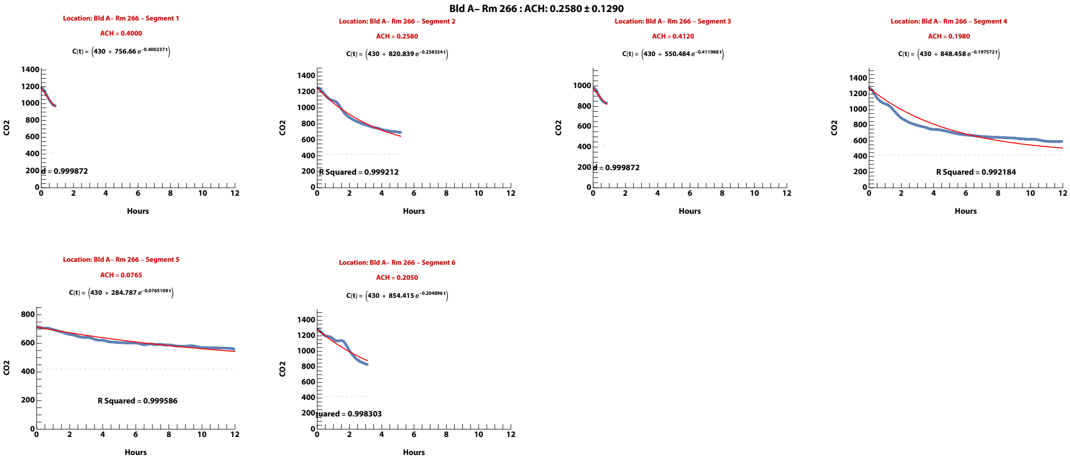

Zand, et al. (2023). Ventilation during COVID–19 in a school for students with intellectual and developmental disabilities (IDD) [Submitted]

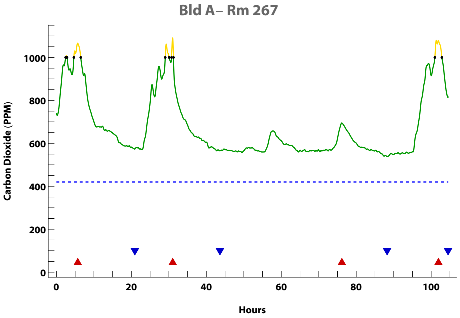

Bld A– Rm 267 : ACH: 0.1520 ± 0.0527

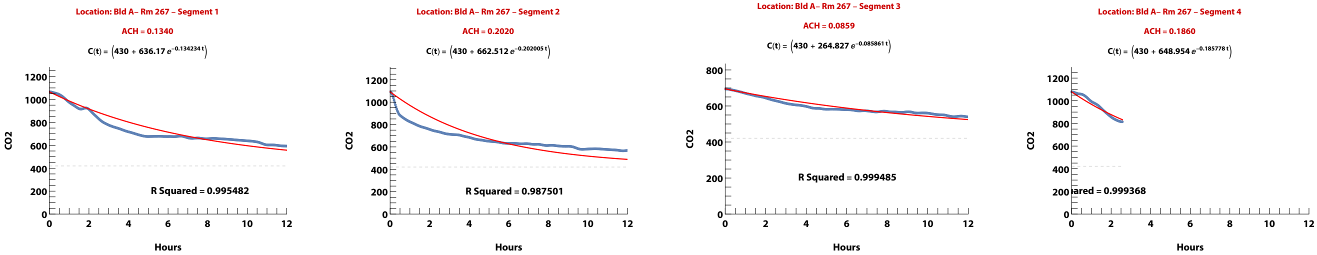

Zand, et al. (2023). Ventilation during COVID–19 in a school for students with intellectual and developmental disabilities (IDD) [Submitted]

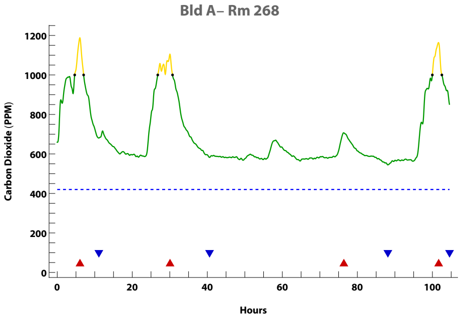

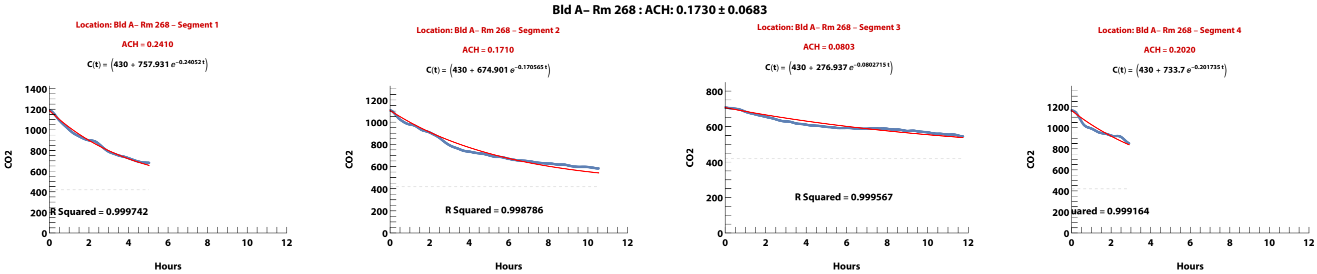

Zand, et al. (2023). Ventilation during COVID–19 in a school for students with intellectual and developmental disabilities (IDD) [Submitted]

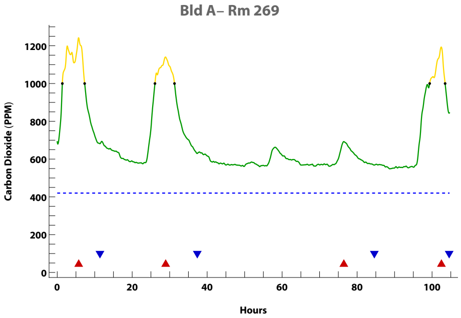

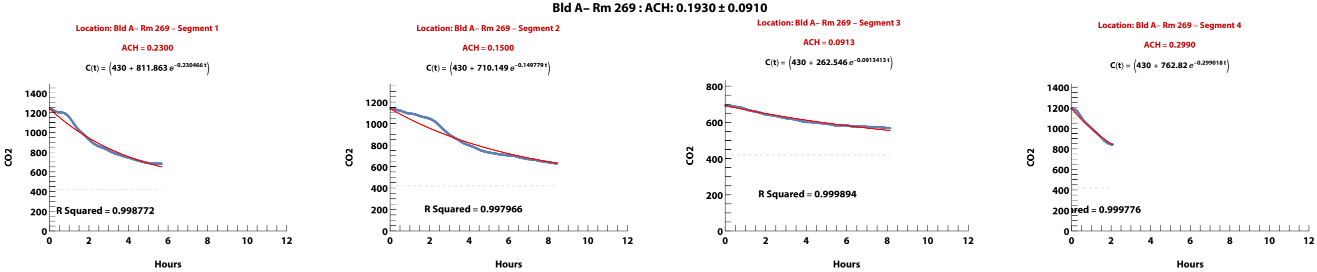

Zand, et al. (2023). Ventilation during COVID–19 in a school for students with intellectual and developmental disabilities (IDD) [Submitted]

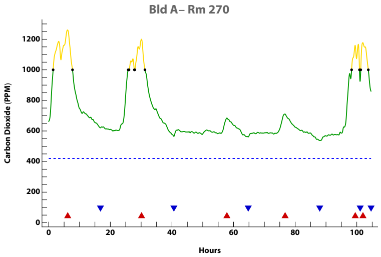

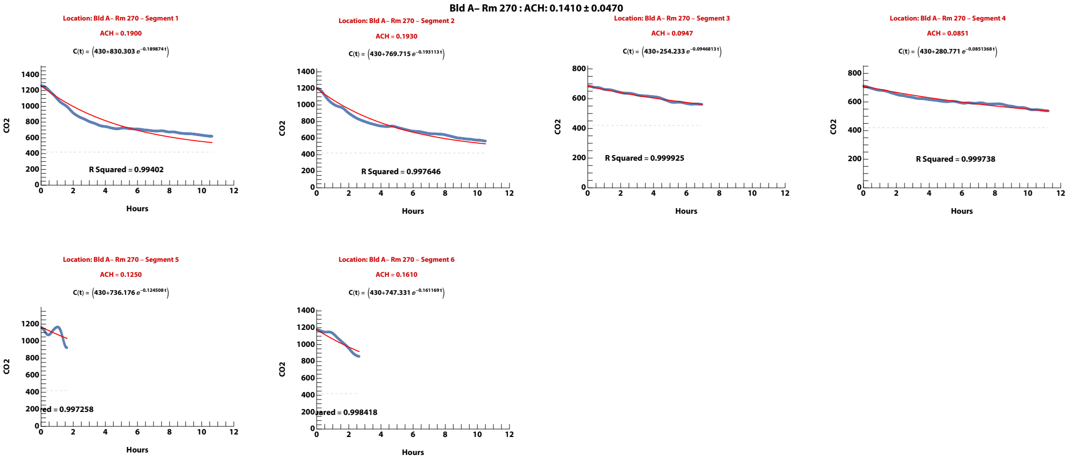

Zand, et al. (2023). Ventilation during COVID–19 in a school for students with intellectual and developmental disabilities (IDD) [Submitted]

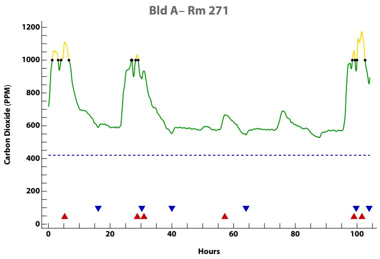

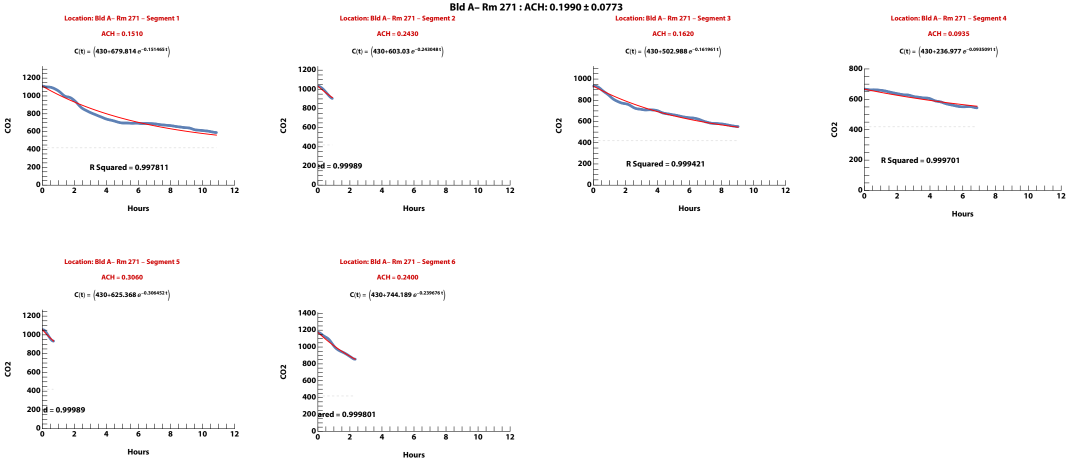

Zand, et al. (2023). Ventilation during COVID–19 in a school for students with intellectual and developmental disabilities (IDD) [Submitted]

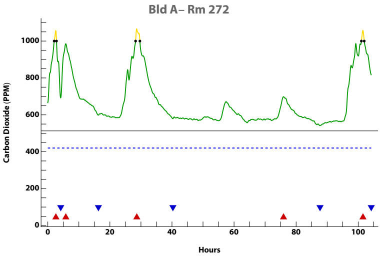

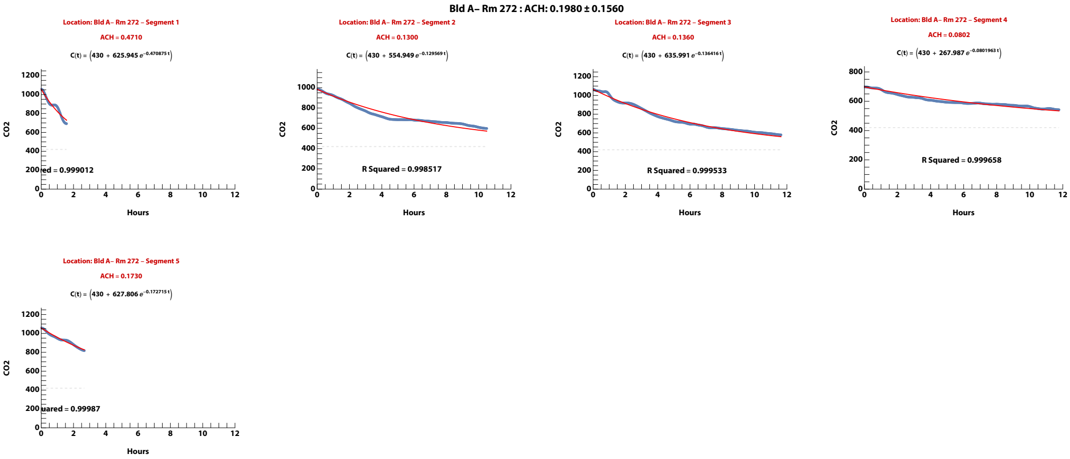

Zand, et al. (2023). Ventilation during COVID–19 in a school for students with intellectual and developmental disabilities (IDD) [Submitted]

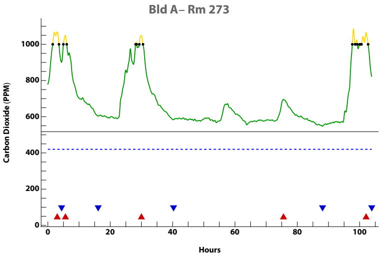

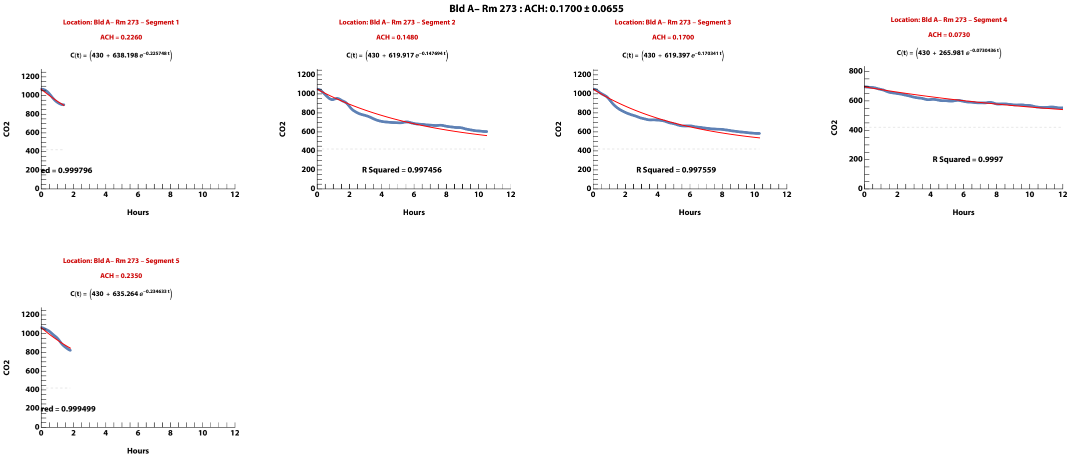

Zand, et al. (2023). Ventilation during COVID–19 in a school for students with intellectual and developmental disabilities (IDD) [Submitted]

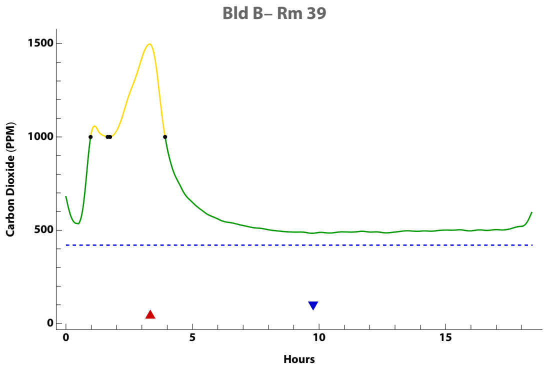

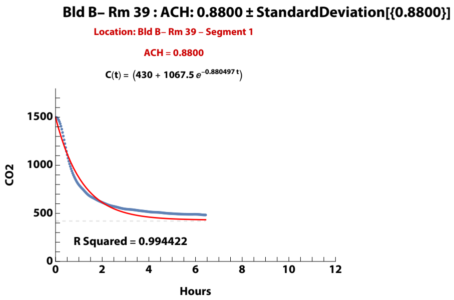

Zand, et al. (2023). Ventilation during COVID–19 in a school for students with intellectual and developmental disabilities (IDD) [Submitted]

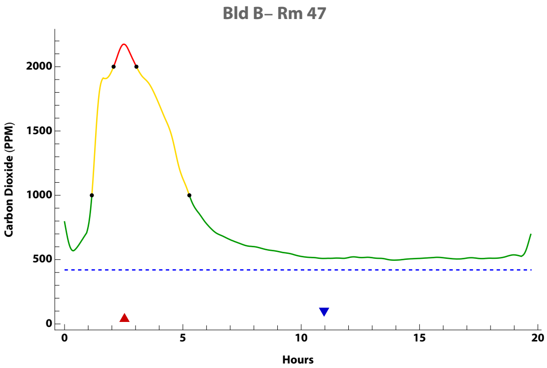

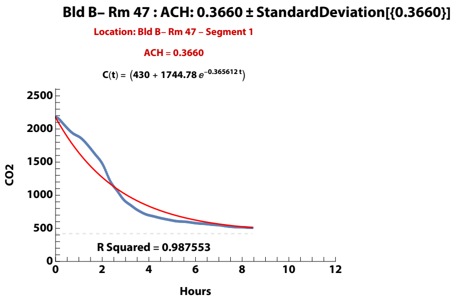

Zand, et al. (2023). Ventilation during COVID–19 in a school for students with intellectual and developmental disabilities (IDD) [Submitted]

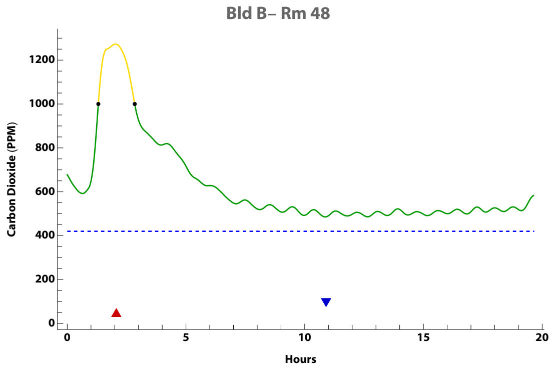

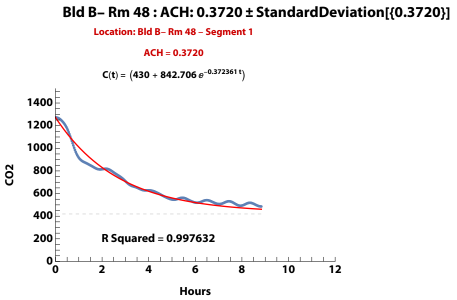

Zand, et al. (2023). Ventilation during COVID–19 in a school for students with intellectual and developmental disabilities (IDD) [Submitted]

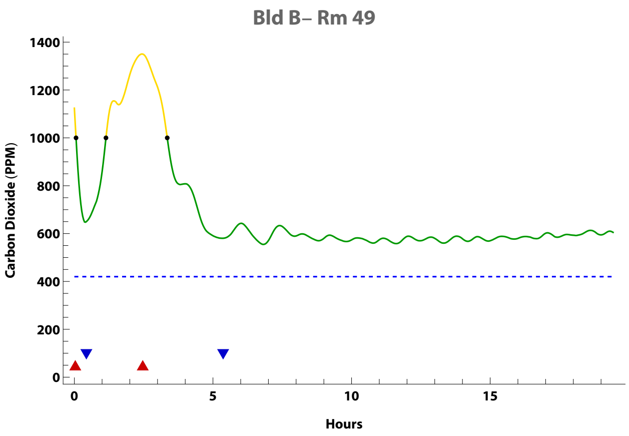

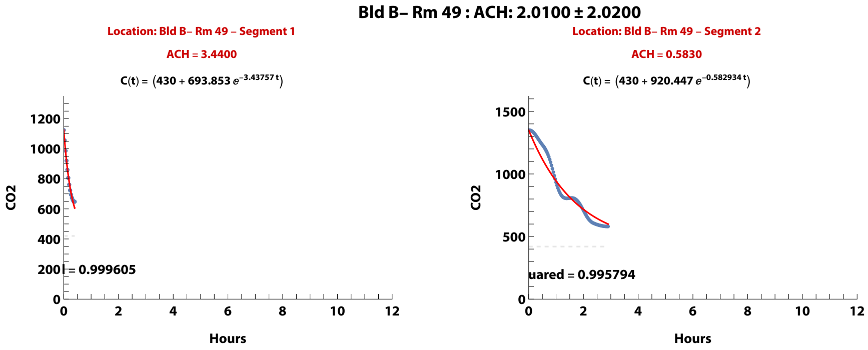

Zand, et al. (2023). Ventilation during COVID–19 in a school for students with intellectual and developmental disabilities (IDD) [Submitted]

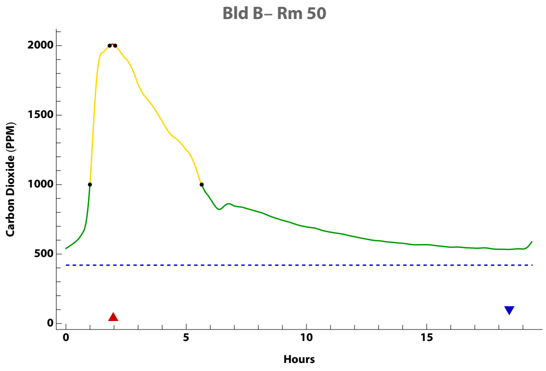

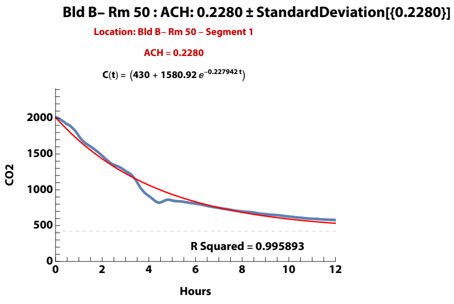

Zand, et al. (2023). Ventilation during COVID–19 in a school for students with intellectual and developmental disabilities (IDD) [Submitted]

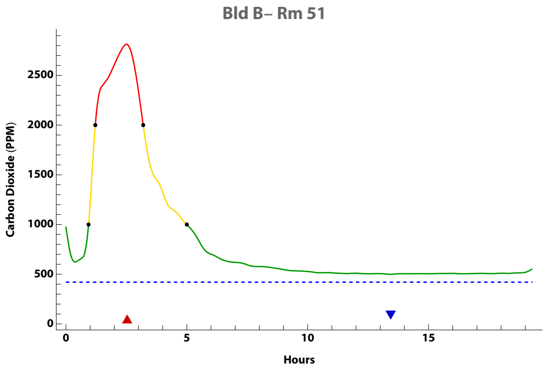

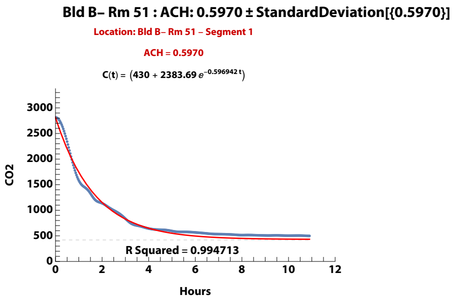

Zand, et al. (2023). Ventilation during COVID–19 in a school for students with intellectual and developmental disabilities (IDD) [Submitted]

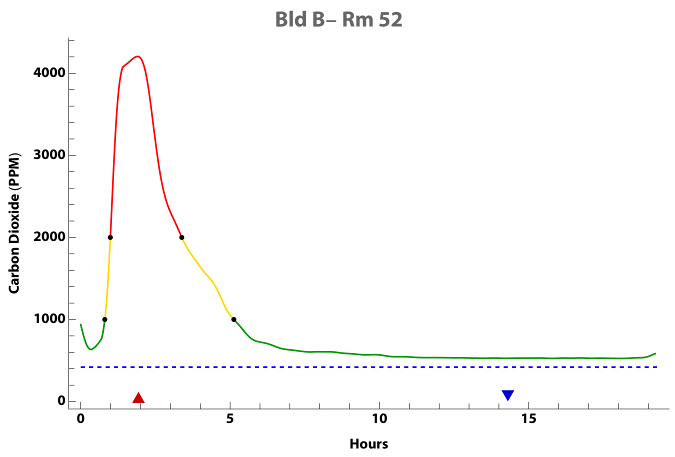

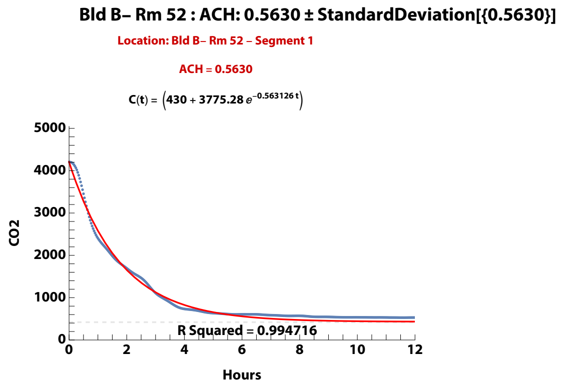

Zand, et al. (2023). Ventilation during COVID–19 in a school for students with intellectual and developmental disabilities (IDD) [Submitted]

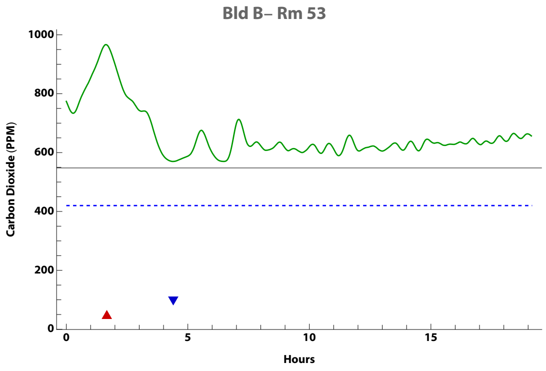

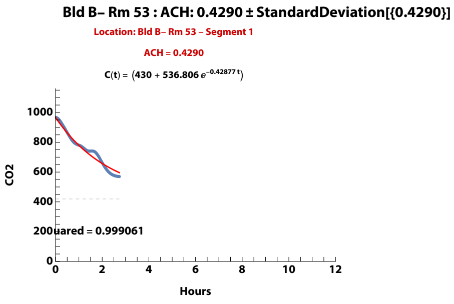

Zand, et al. (2023). Ventilation during COVID–19 in a school for students with intellectual and developmental disabilities (IDD) [Submitted]

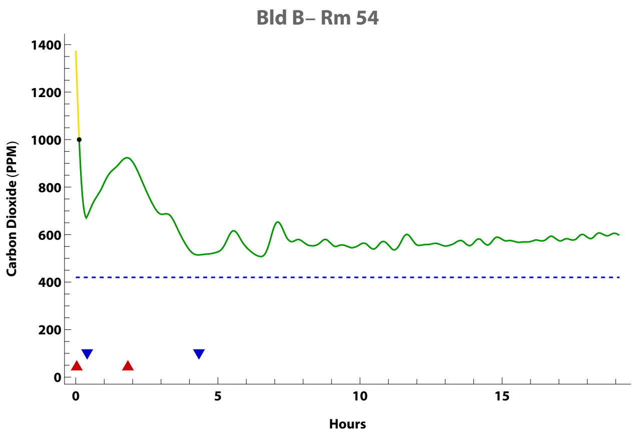

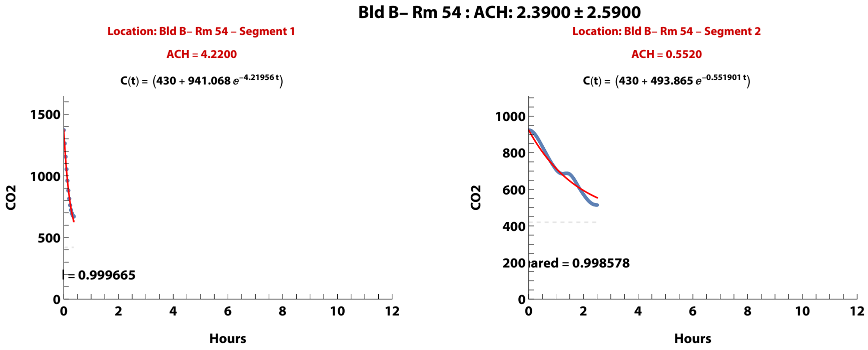

Zand, et al. (2023). Ventilation during COVID–19 in a school for students with intellectual and developmental disabilities (IDD) [Submitted]

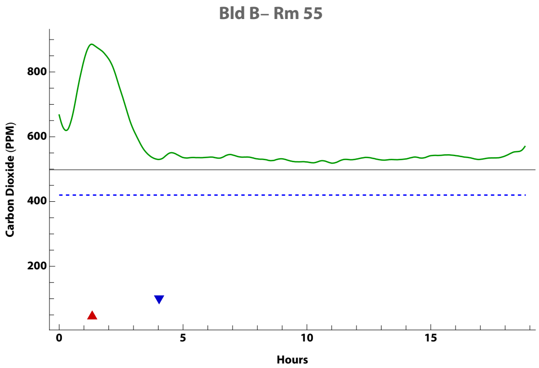

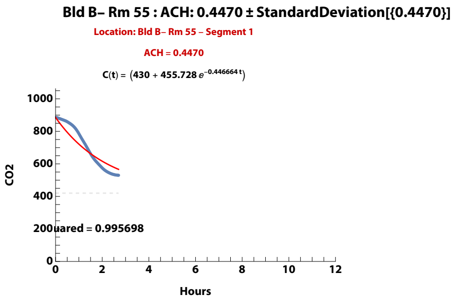

Zand, et al. (2023). Ventilation during COVID–19 in a school for students with intellectual and developmental disabilities (IDD) [Submitted]

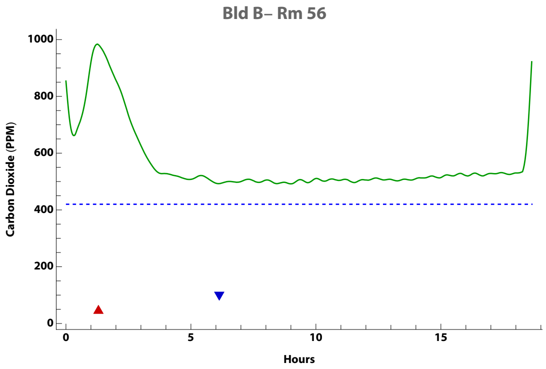

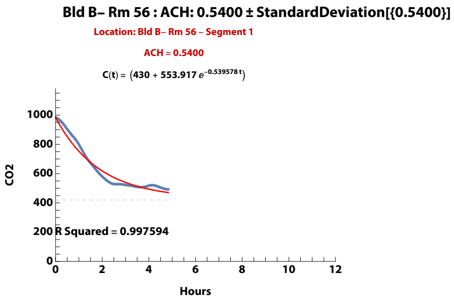

Zand, et al. (2023). Ventilation during COVID–19 in a school for students with intellectual and developmental disabilities (IDD) [Submitted]

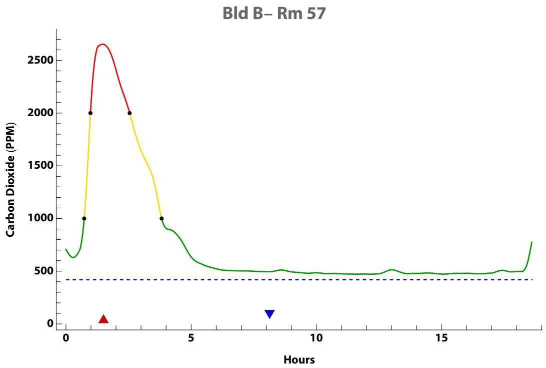

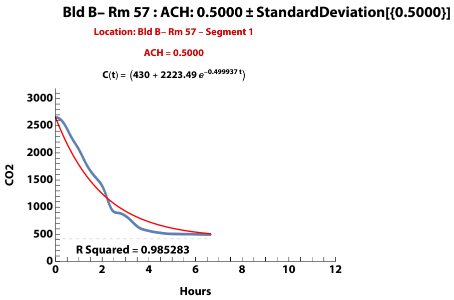

Zand, et al. (2023). Ventilation during COVID–19 in a school for students with intellectual and developmental disabilities (IDD) [Submitted]

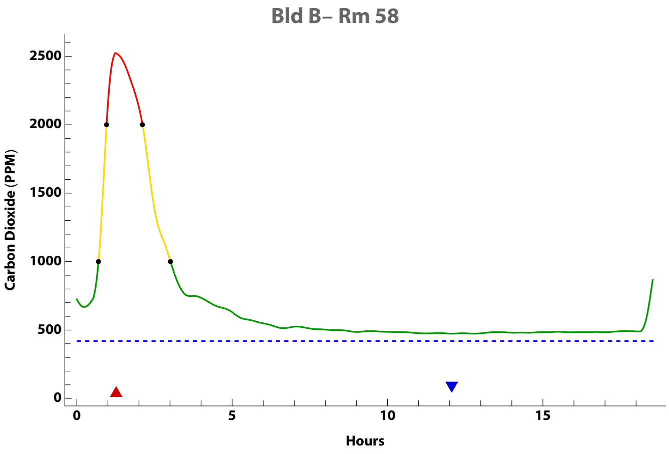

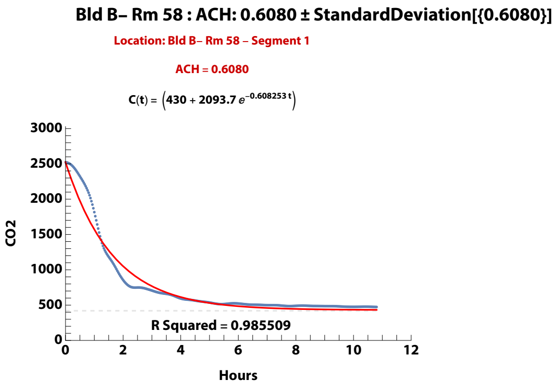

Zand, et al. (2023). Ventilation during COVID–19 in a school for students with intellectual and developmental disabilities (IDD) [Submitted]

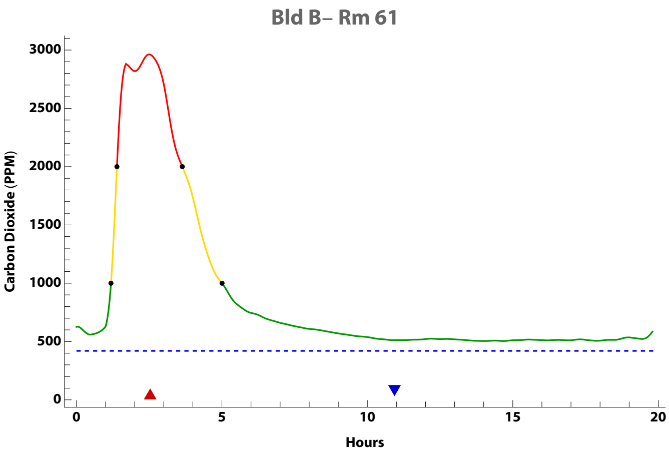

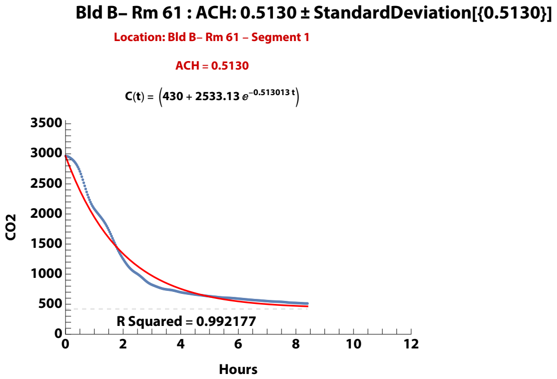

Zand, et al. (2023). Ventilation during COVID–19 in a school for students with intellectual and developmental disabilities (IDD) [Submitted]

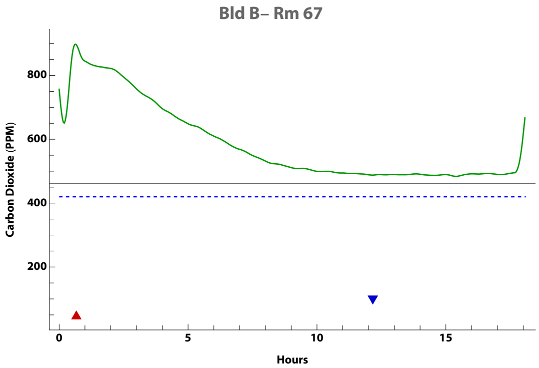

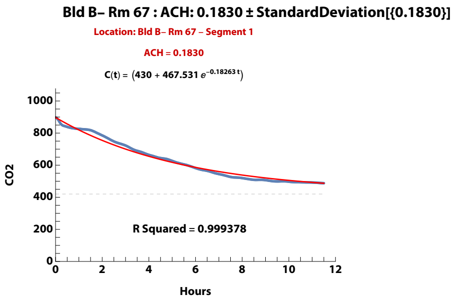

Zand, et al. (2023). Ventilation during COVID–19 in a school for students with intellectual and developmental disabilities (IDD) [Submitted]

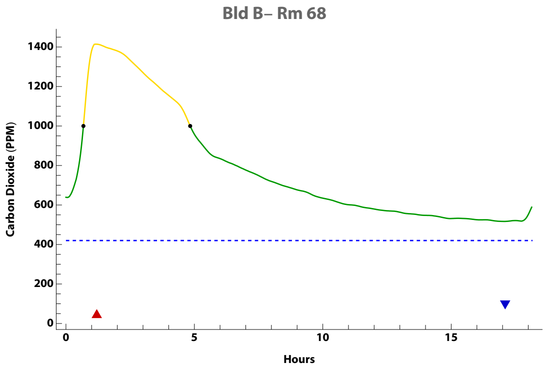

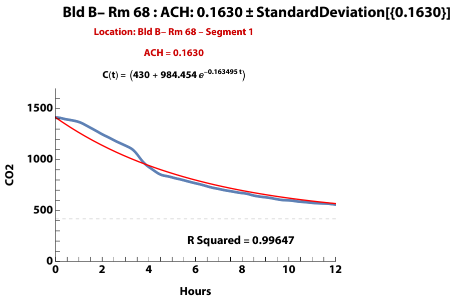

Zand, et al. (2023). Ventilation during COVID–19 in a school for students with intellectual and developmental disabilities (IDD) [Submitted]

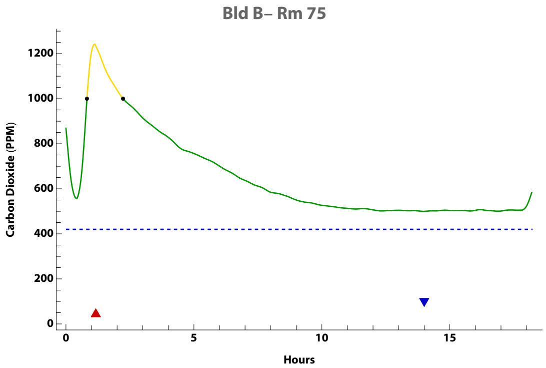

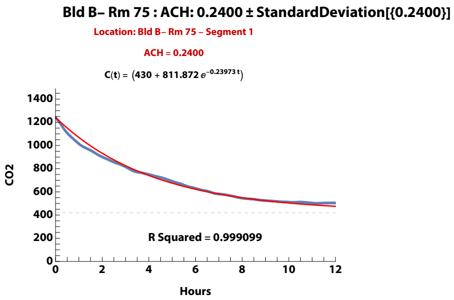

Zand, et al. (2023). Ventilation during COVID–19 in a school for students with intellectual and developmental disabilities (IDD) [Submitted]

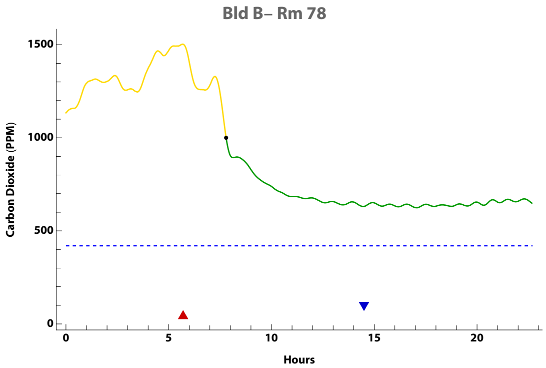

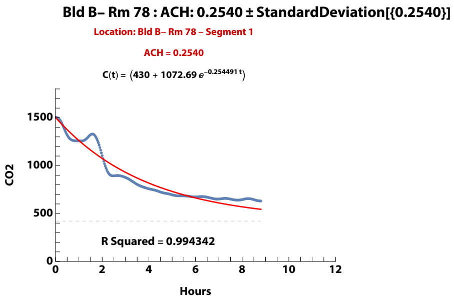

Zand, et al. (2023). Ventilation during COVID–19 in a school for students with intellectual and developmental disabilities (IDD) [Submitted]

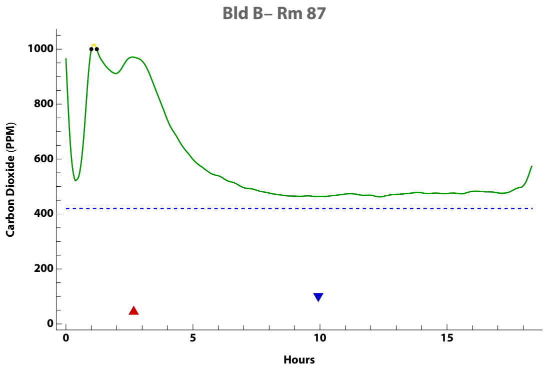

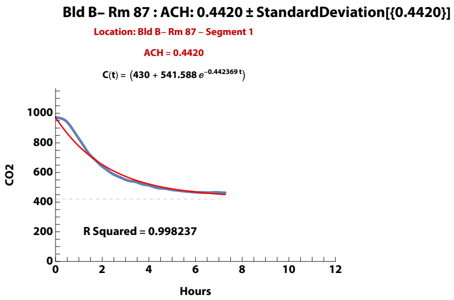

Zand, et al. (2023). Ventilation during COVID–19 in a school for students with intellectual and developmental disabilities (IDD) [Submitted]

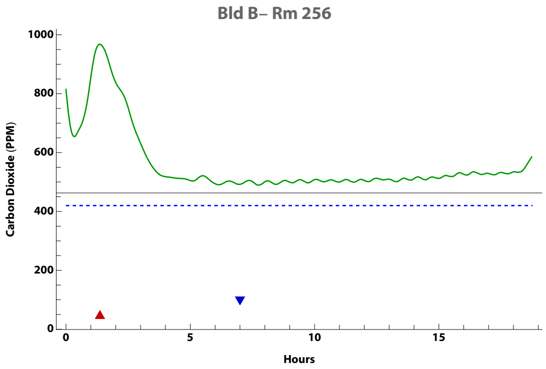

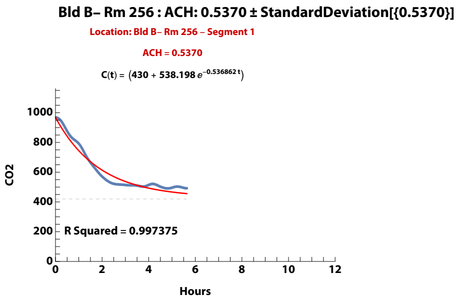

Zand, et al. (2023). Ventilation during COVID–19 in a school for students with intellectual and developmental disabilities (IDD) [Submitted]

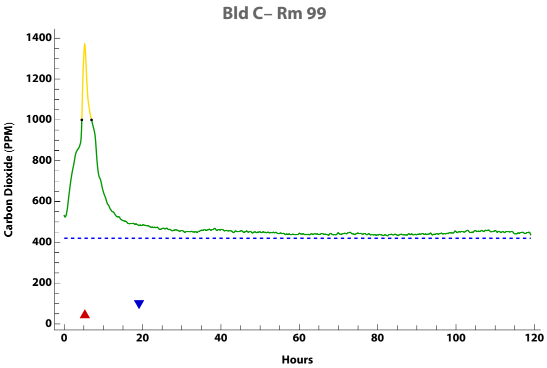

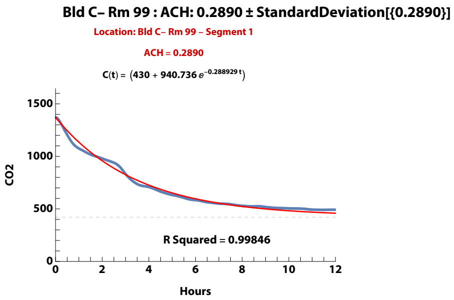

Zand, et al. (2023). Ventilation during COVID–19 in a school for students with intellectual and developmental disabilities (IDD) [Submitted]

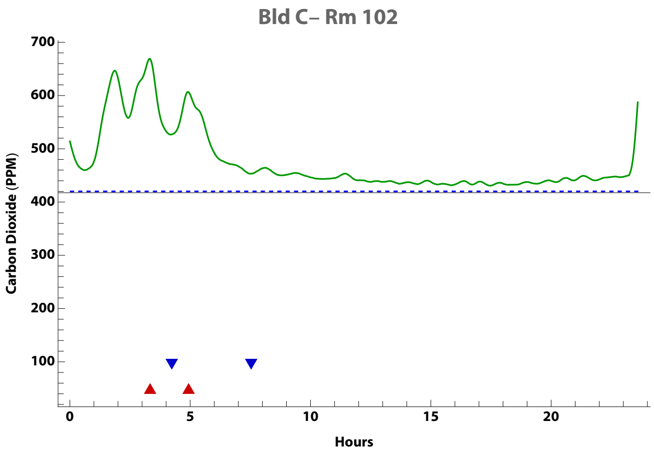

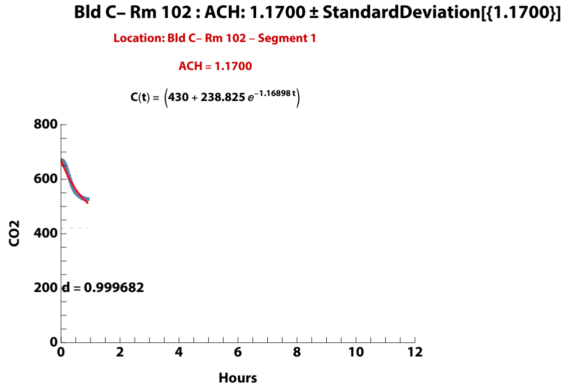

Zand, et al. (2023). Ventilation during COVID–19 in a school for students with intellectual and developmental disabilities (IDD) [Submitted]

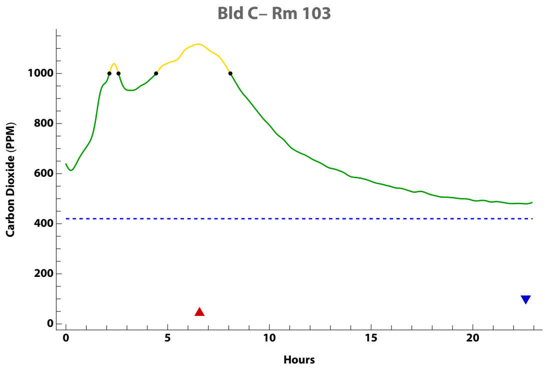

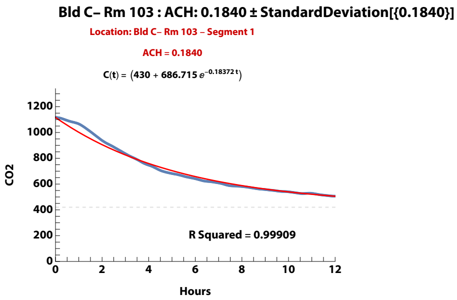

Zand, et al. (2023). Ventilation during COVID–19 in a school for students with intellectual and developmental disabilities (IDD) [Submitted]

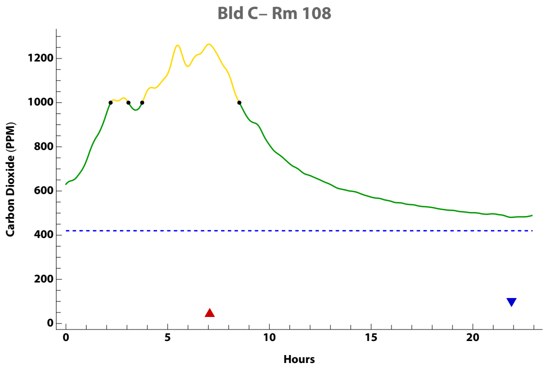

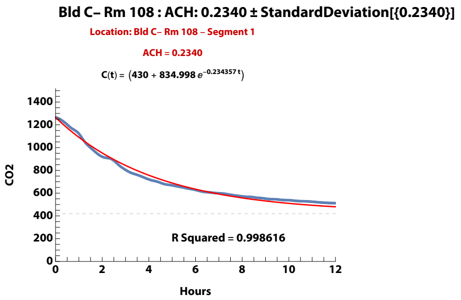

Zand, et al. (2023). Ventilation during COVID–19 in a school for students with intellectual and developmental disabilities (IDD) [Submitted]

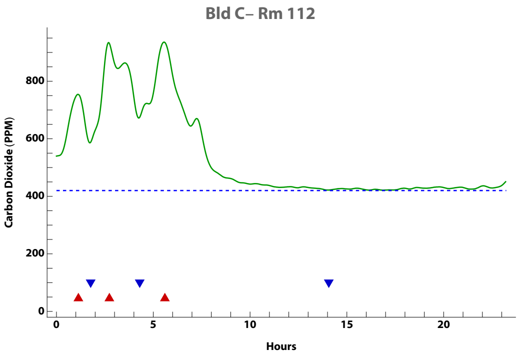

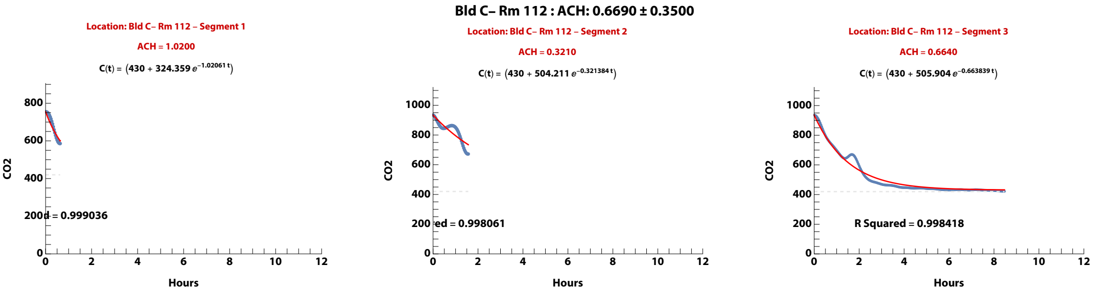

Zand, et al. (2023). Ventilation during COVID–19 in a school for students with intellectual and developmental disabilities (IDD) [Submitted]

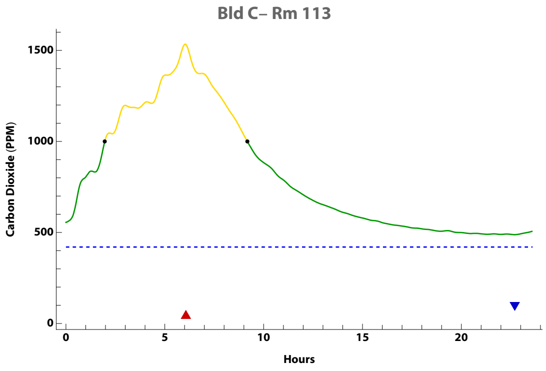

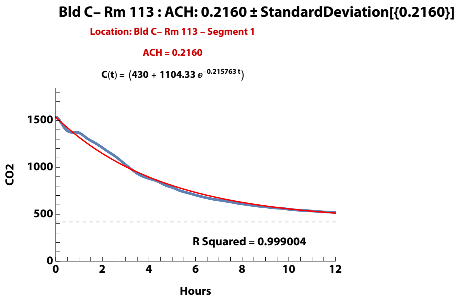

Zand, et al. (2023). Ventilation during COVID–19 in a school for students with intellectual and developmental disabilities (IDD) [Submitted]

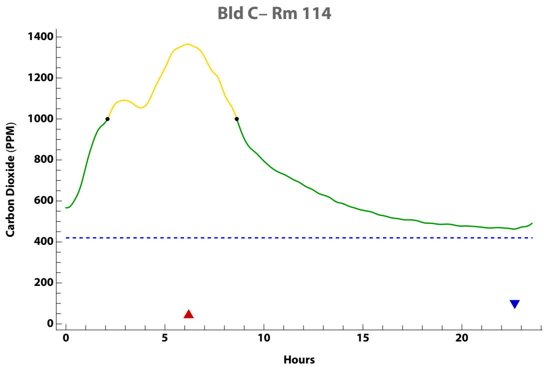

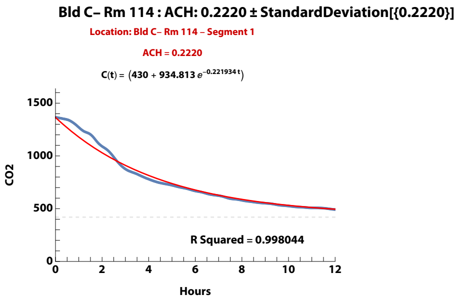

Zand, et al. (2023). Ventilation during COVID–19 in a school for students with intellectual and developmental disabilities (IDD) [Submitted]

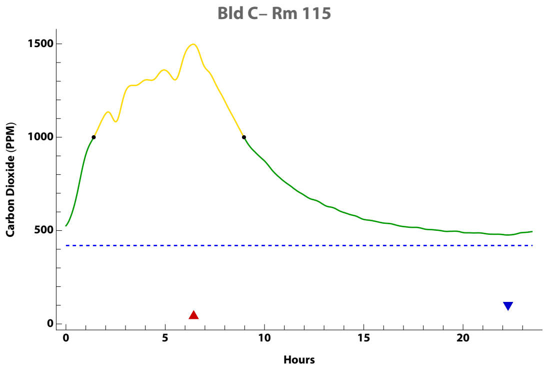

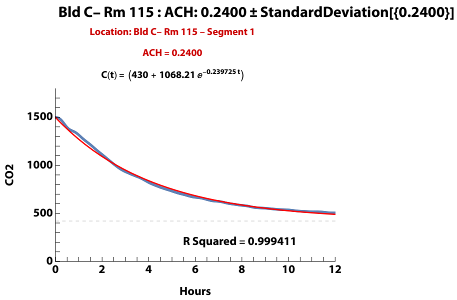

Zand, et al. (2023). Ventilation during COVID–19 in a school for students with intellectual and developmental disabilities (IDD) [Submitted]

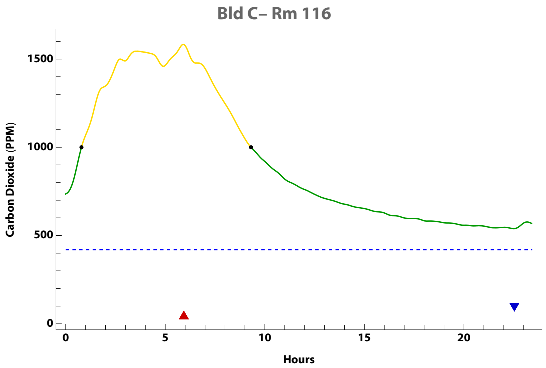

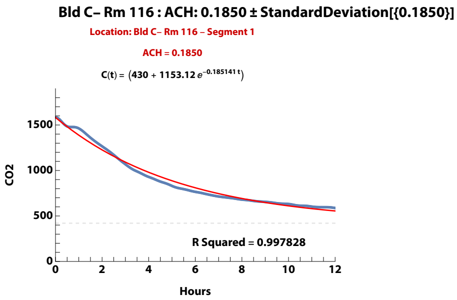

Zand, et al. (2023). Ventilation during COVID–19 in a school for students with intellectual and developmental disabilities (IDD) [Submitted]

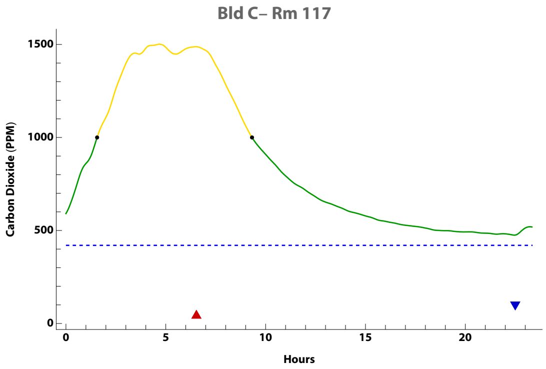

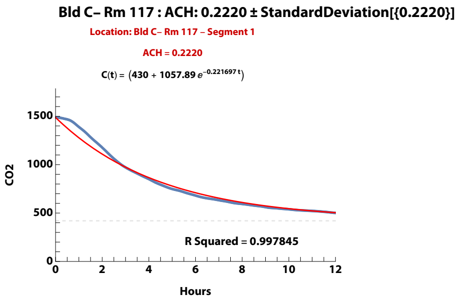

Zand, et al. (2023). Ventilation during COVID–19 in a school for students with intellectual and developmental disabilities (IDD) [Submitted]

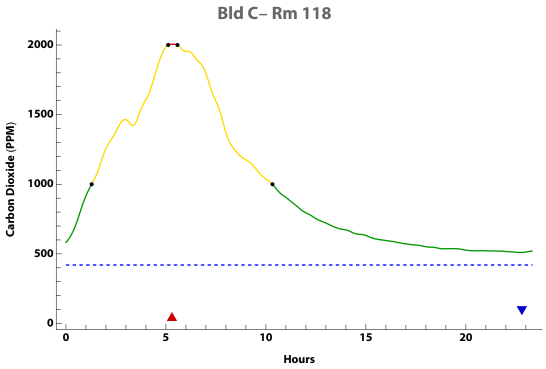

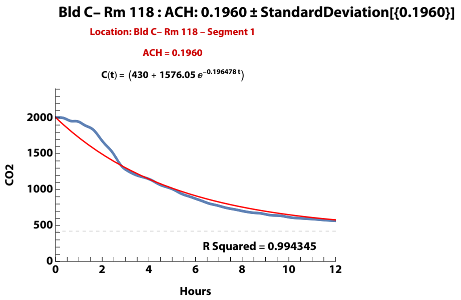

Zand, et al. (2023). Ventilation during COVID–19 in a school for students with intellectual and developmental disabilities (IDD) [Submitted]

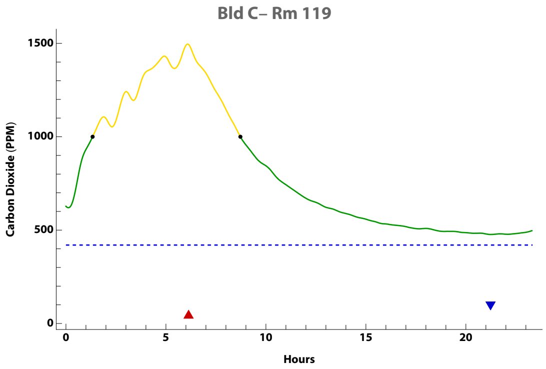

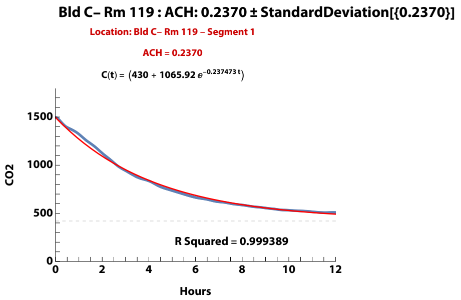

Zand, et al. (2023). Ventilation during COVID–19 in a school for students with intellectual and developmental disabilities (IDD) [Submitted]

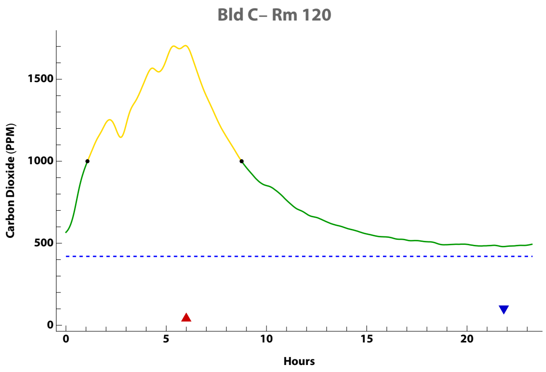

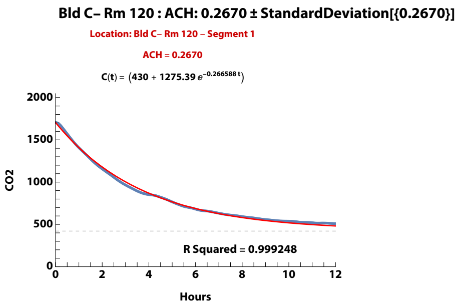

Zand, et al. (2023). Ventilation during COVID–19 in a school for students with intellectual and developmental disabilities (IDD) [Submitted]

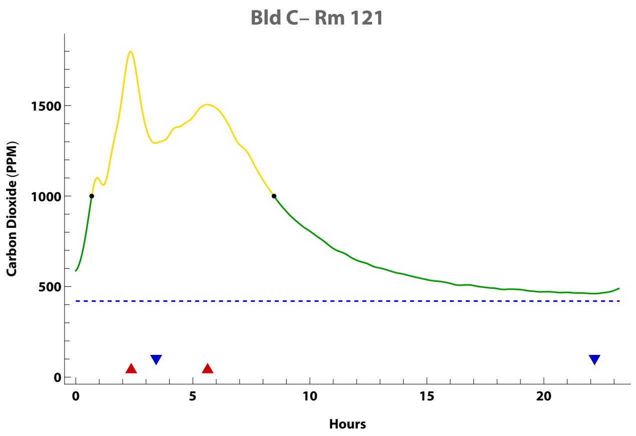

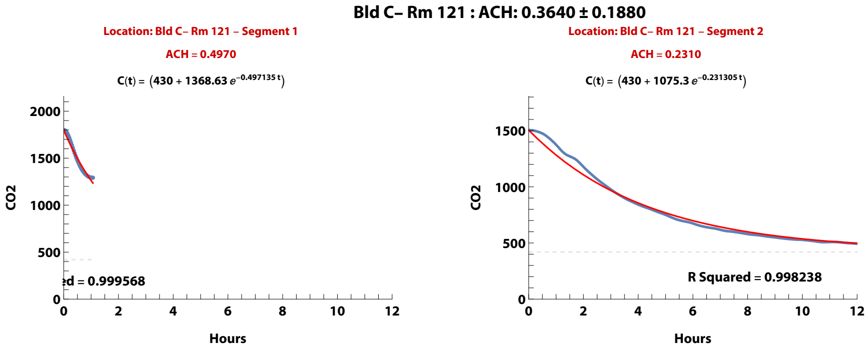

Zand, et al. (2023). Ventilation during COVID–19 in a school for students with intellectual and developmental disabilities (IDD) [Submitted]

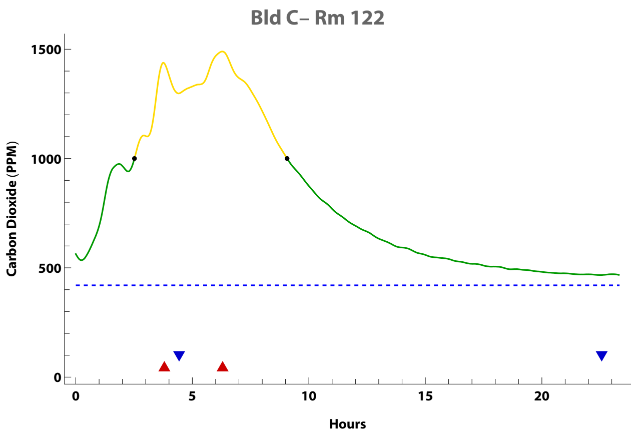

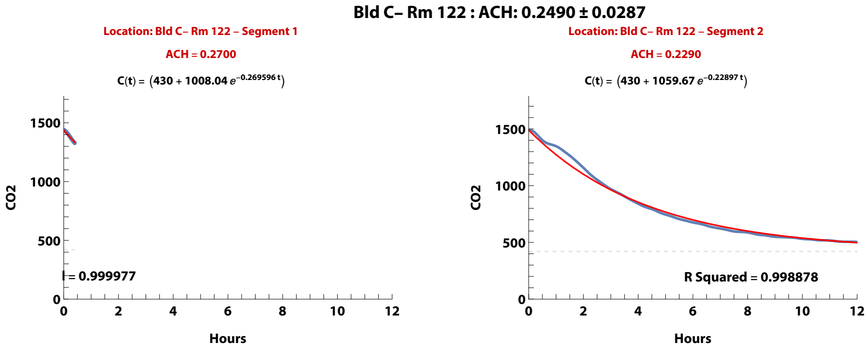

Zand, et al. (2023). Ventilation during COVID–19 in a school for students with intellectual and developmental disabilities (IDD) [Submitted]

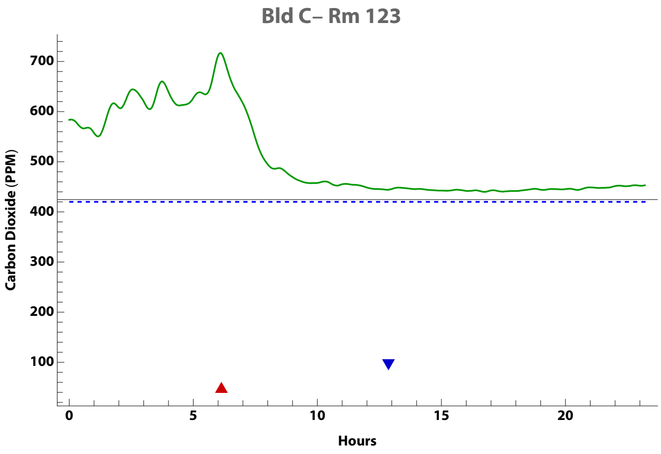

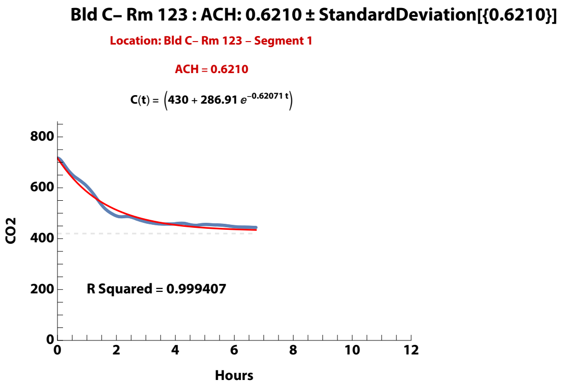

Zand, et al. (2023). Ventilation during COVID–19 in a school for students with intellectual and developmental disabilities (IDD) [Submitted]

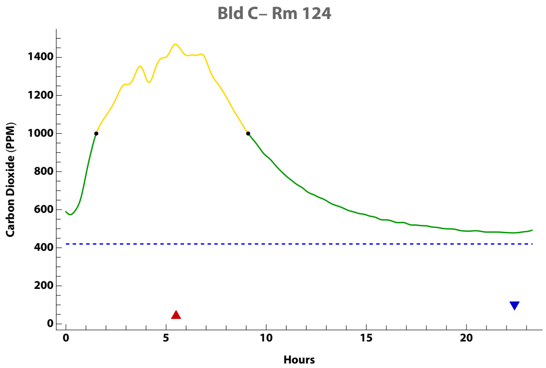

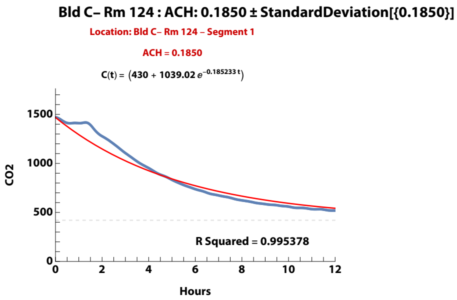

Zand, et al. (2023). Ventilation during COVID–19 in a school for students with intellectual and developmental disabilities (IDD) [Submitted]

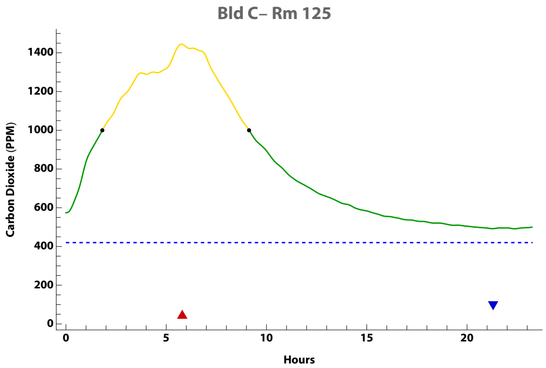

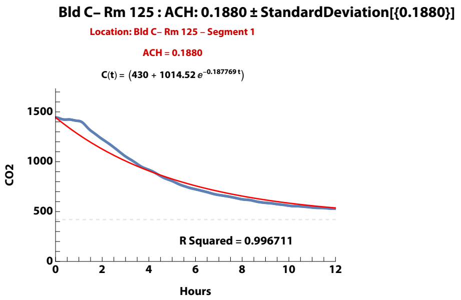

Zand, et al. (2023). Ventilation during COVID–19 in a school for students with intellectual and developmental disabilities (IDD) [Submitted]

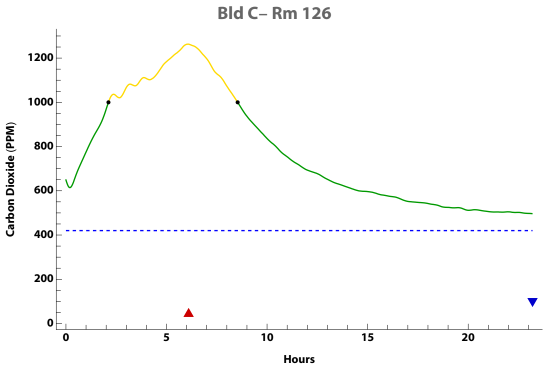

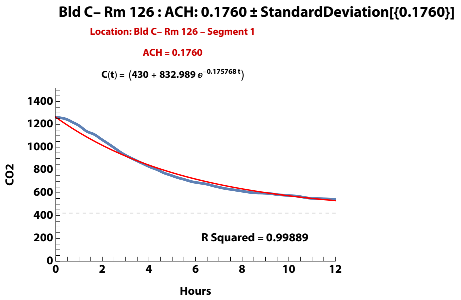

Zand, et al. (2023). Ventilation during COVID–19 in a school for students with intellectual and developmental disabilities (IDD) [Submitted]

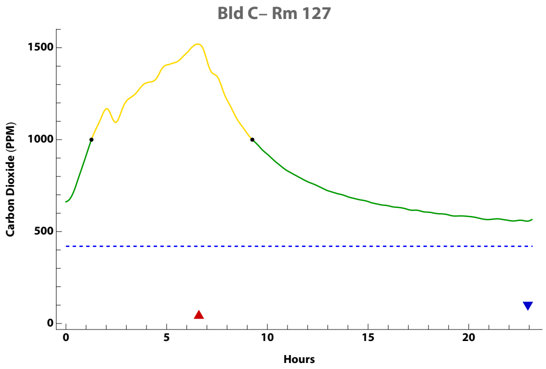

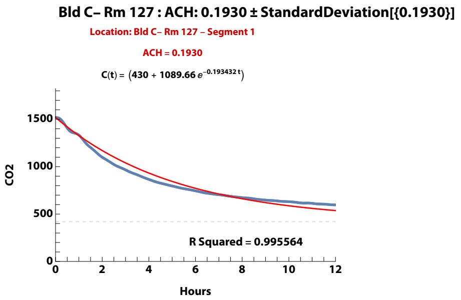

Zand, et al. (2023). Ventilation during COVID–19 in a school for students with intellectual and developmental disabilities (IDD) [Submitted]

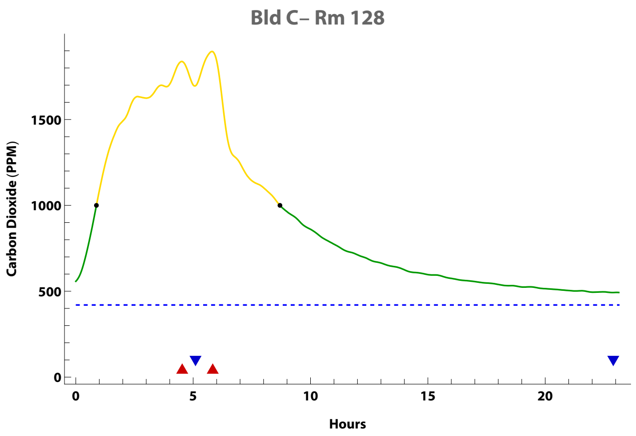

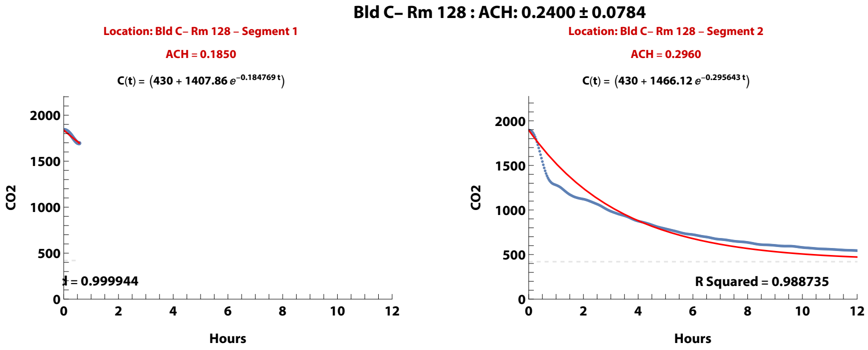

Zand, et al. (2023). Ventilation during COVID–19 in a school for students with intellectual and developmental disabilities (IDD) [Submitted]

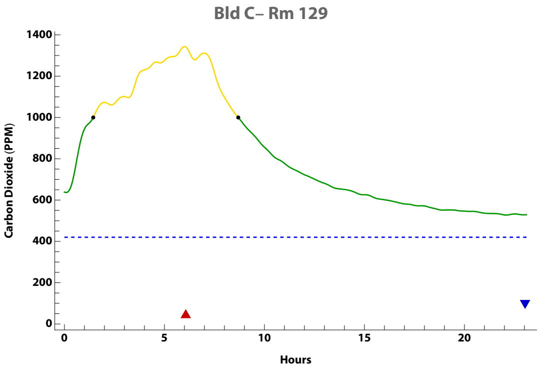

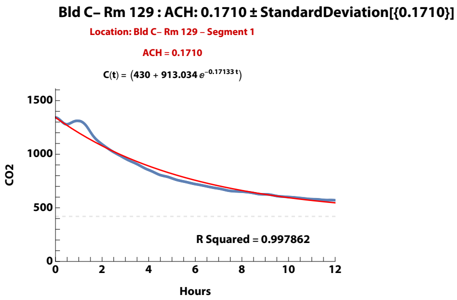

Zand, et al. (2023). Ventilation during COVID–19 in a school for students with intellectual and developmental disabilities (IDD) [Submitted]

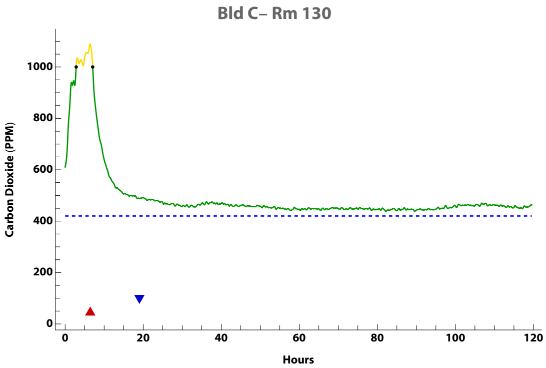

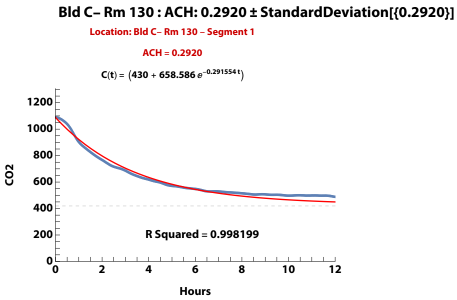

Zand, et al. (2023). Ventilation during COVID–19 in a school for students with intellectual and developmental disabilities (IDD) [Submitted]

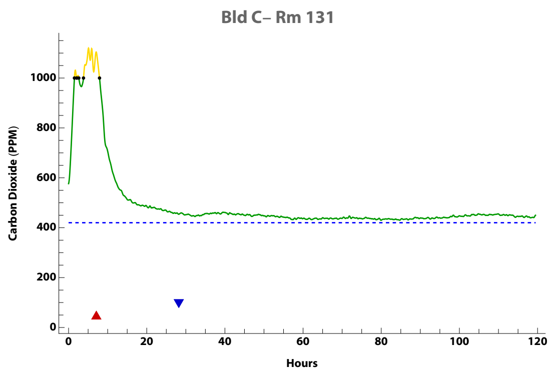

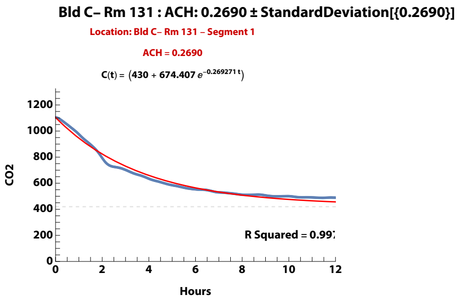

Zand, et al. (2023). Ventilation during COVID–19 in a school for students with intellectual and developmental disabilities (IDD) [Submitted]

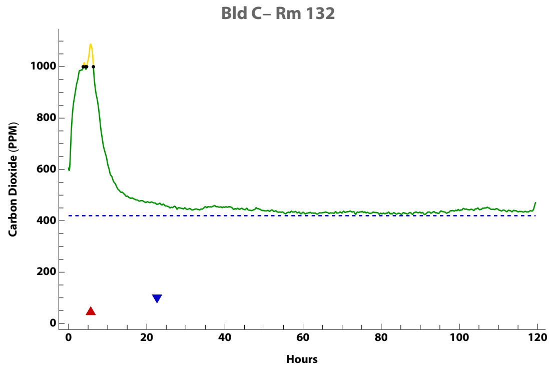

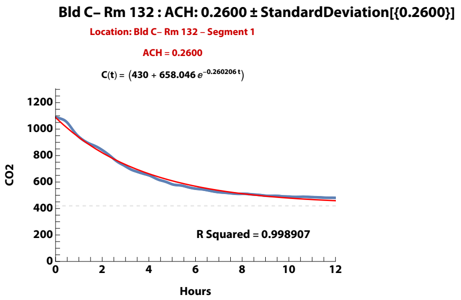

Zand, et al. (2023). Ventilation during COVID–19 in a school for students with intellectual and developmental disabilities (IDD) [Submitted]

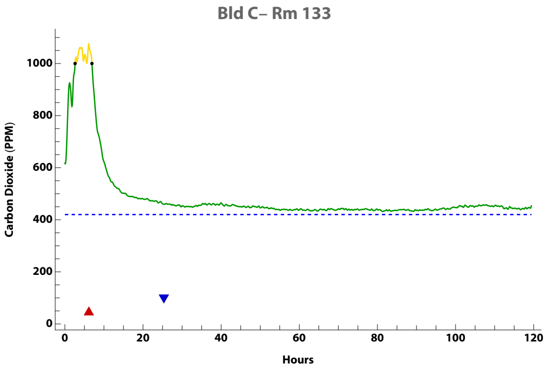

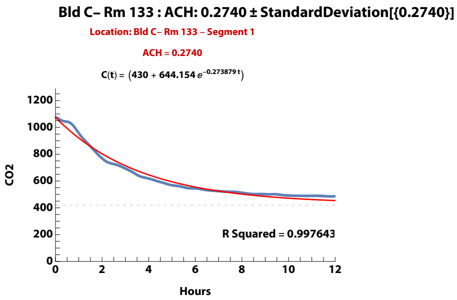

Zand, et al. (2023). Ventilation during COVID–19 in a school for students with intellectual and developmental disabilities (IDD) [Submitted]

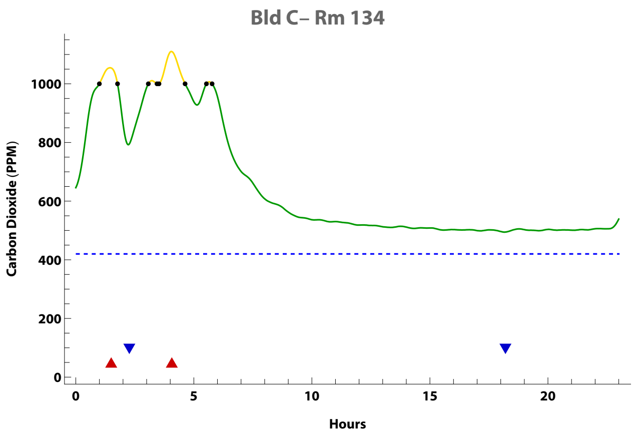

Bld C– Rm 134 : ACH: 0.4430 ± 0.2530

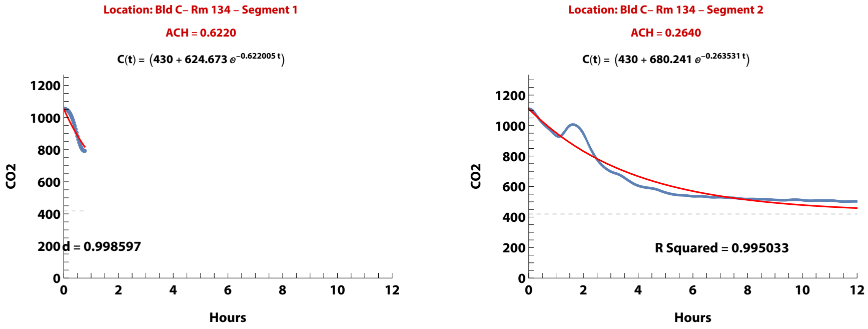

Zand, et al. (2023). Ventilation during COVID–19 in a school for students with intellectual and developmental disabilities (IDD) [Submitted]

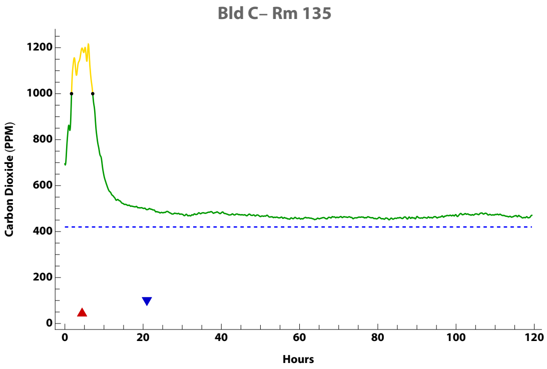

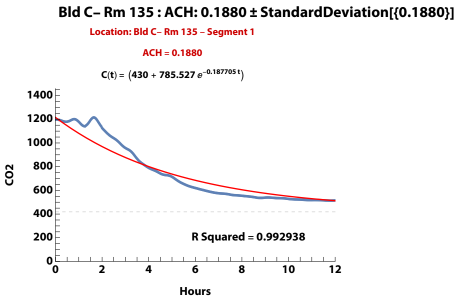

Zand, et al. (2023). Ventilation during COVID–19 in a school for students with intellectual and developmental disabilities (IDD) [Submitted]

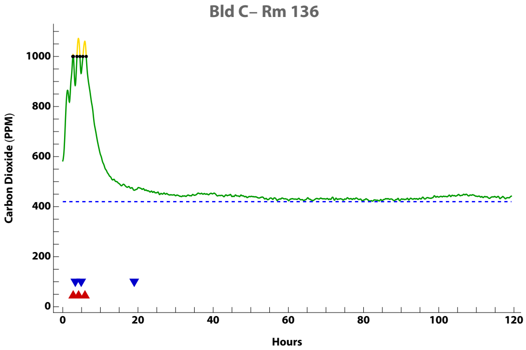

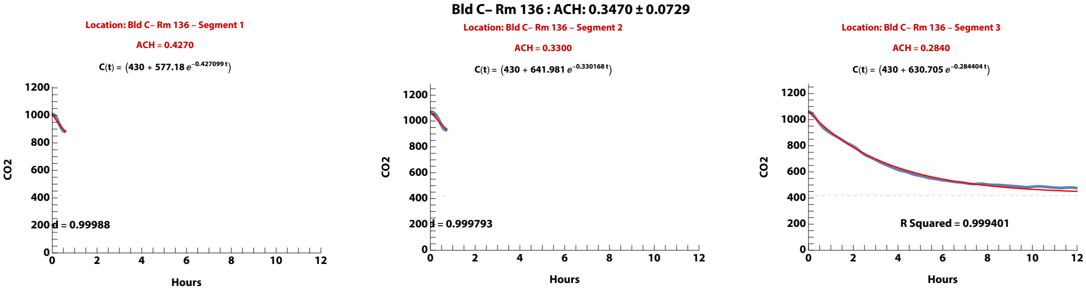

Zand, et al. (2023). Ventilation during COVID–19 in a school for students with intellectual and developmental disabilities (IDD) [Submitted]

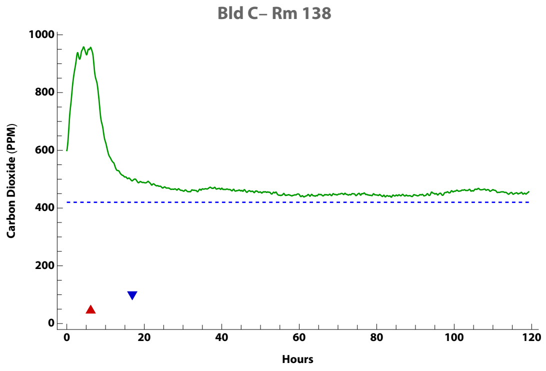

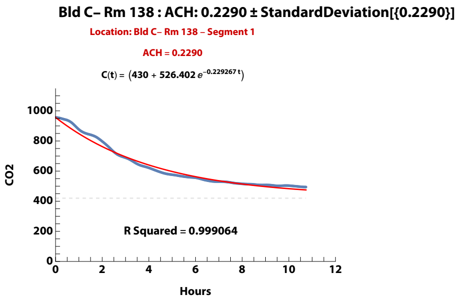

Zand, et al. (2023). Ventilation during COVID–19 in a school for students with intellectual and developmental disabilities (IDD) [Submitted]

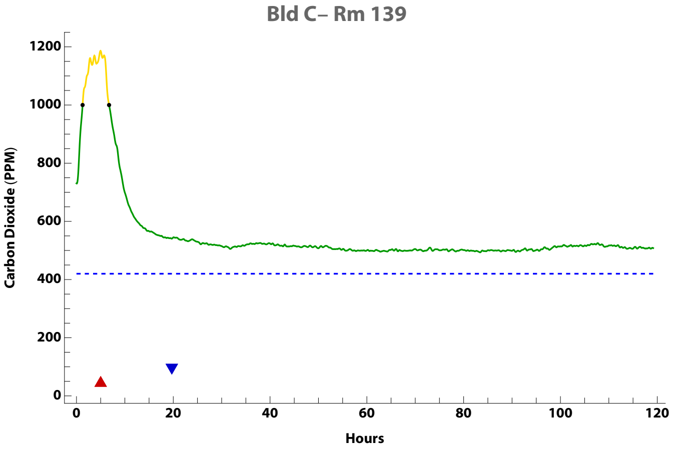

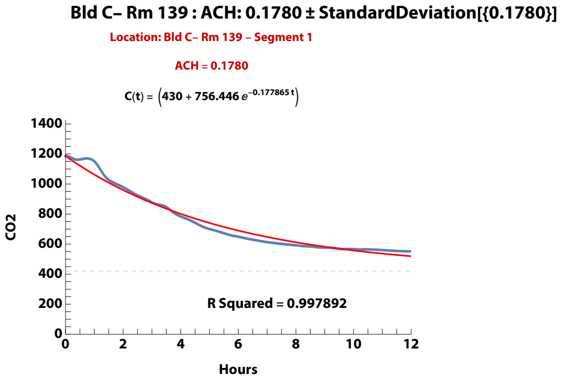

Zand, et al. (2023). Ventilation during COVID–19 in a school for students with intellectual and developmental disabilities (IDD) [Submitted]

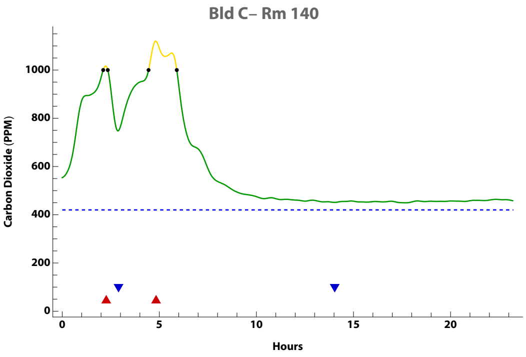

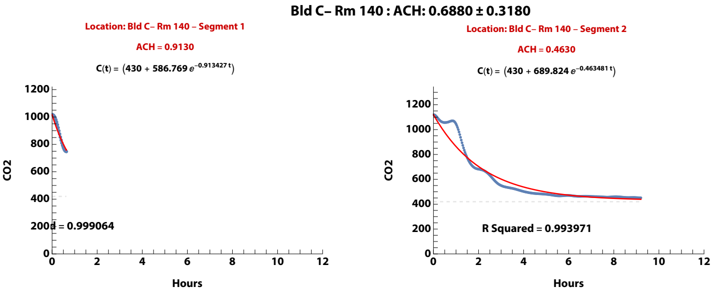

Zand, et al. (2023). Ventilation during COVID–19 in a school for students with intellectual and developmental disabilities (IDD) [Submitted]

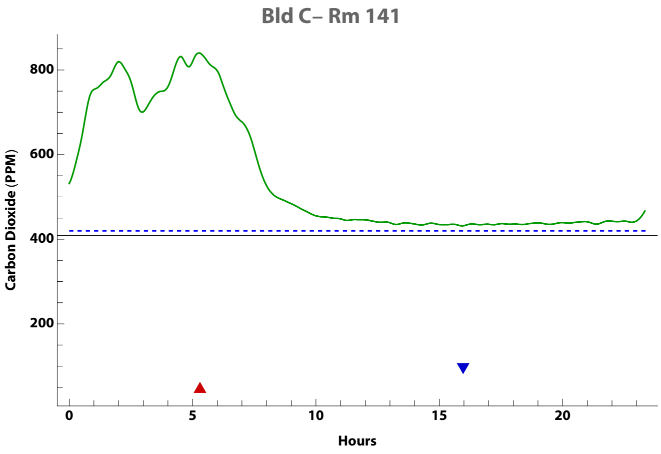

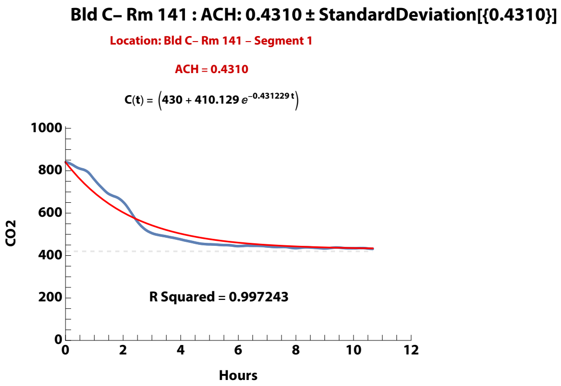

Zand, et al. (2023). Ventilation during COVID–19 in a school for students with intellectual and developmental disabilities (IDD) [Submitted]

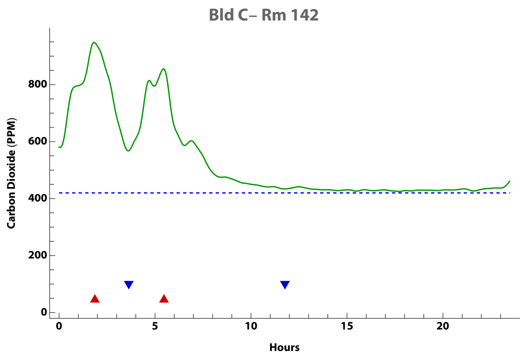

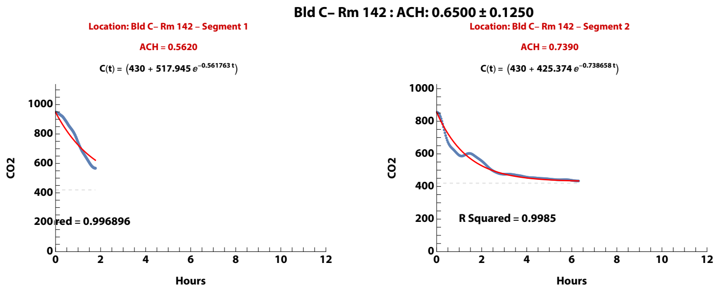

Zand, et al. (2023). Ventilation during COVID–19 in a school for students with intellectual and developmental disabilities (IDD) [Submitted]

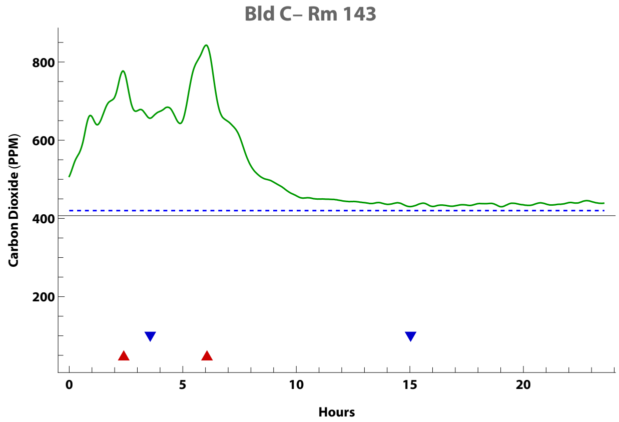

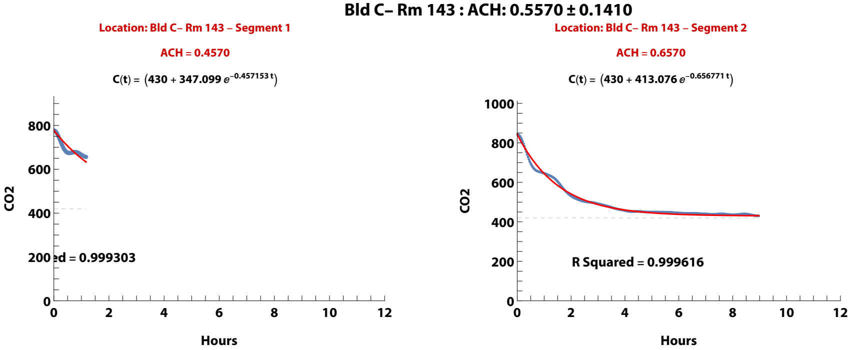

Zand, et al. (2023). Ventilation during COVID–19 in a school for students with intellectual and developmental disabilities (IDD) [Submitted]

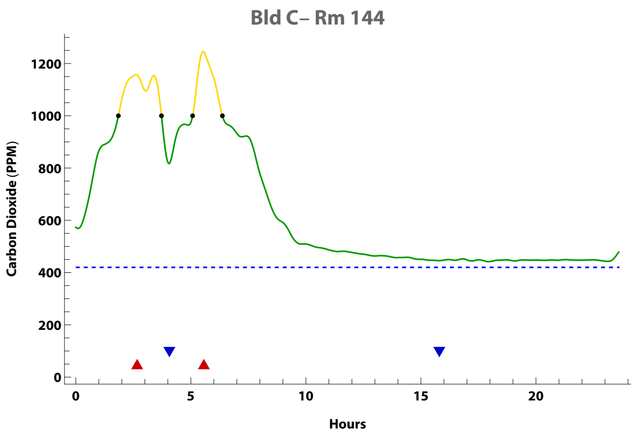

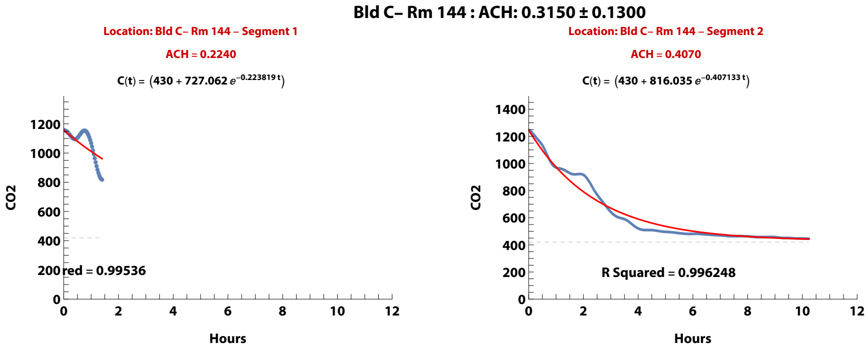

Zand, et al. (2023). Ventilation during COVID–19 in a school for students with intellectual and developmental disabilities (IDD) [Submitted]

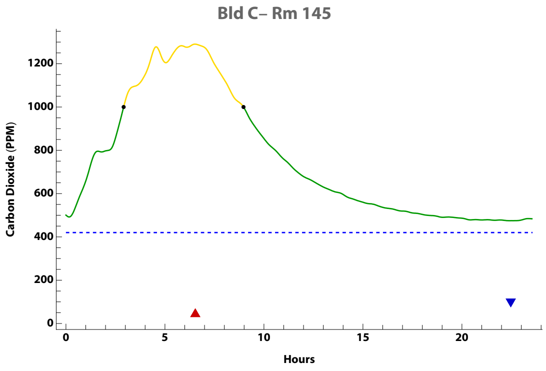

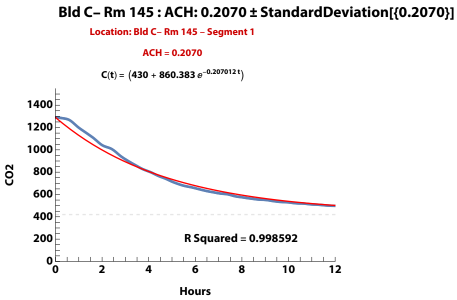

Zand, et al. (2023). Ventilation during COVID–19 in a school for students with intellectual and developmental disabilities (IDD) [Submitted]

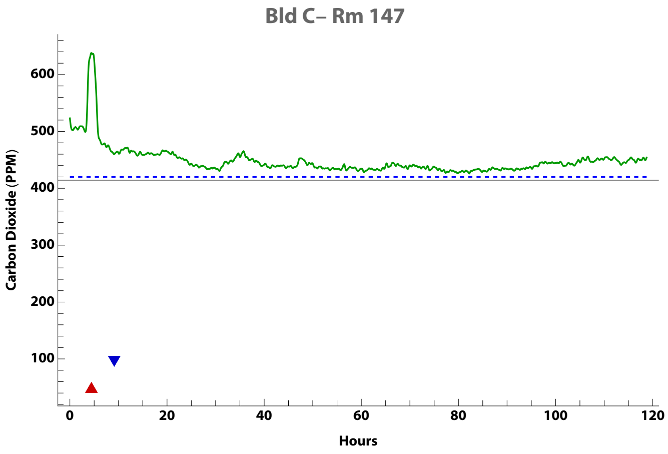

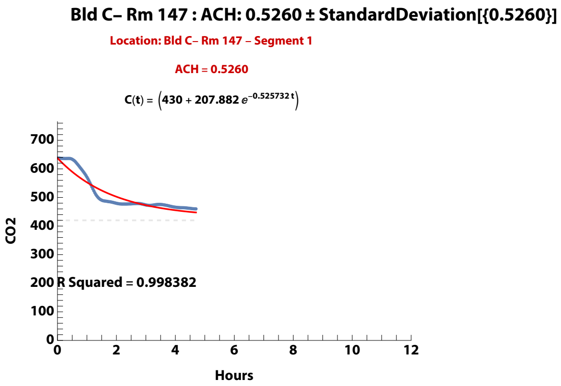

Zand, et al. (2023). Ventilation during COVID–19 in a school for students with intellectual and developmental disabilities (IDD) [Submitted]

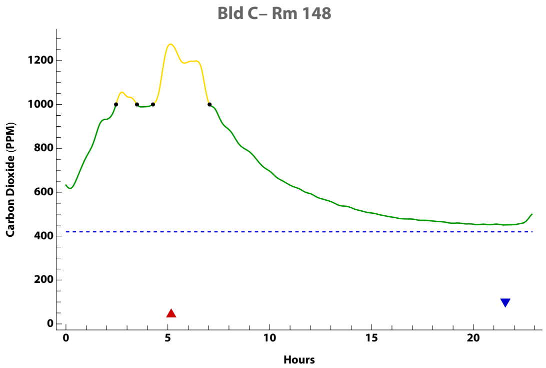

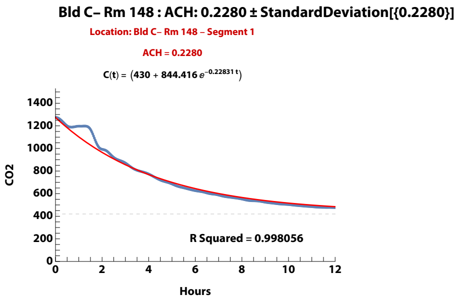

Zand, et al. (2023). Ventilation during COVID–19 in a school for students with intellectual and developmental disabilities (IDD) [Submitted]

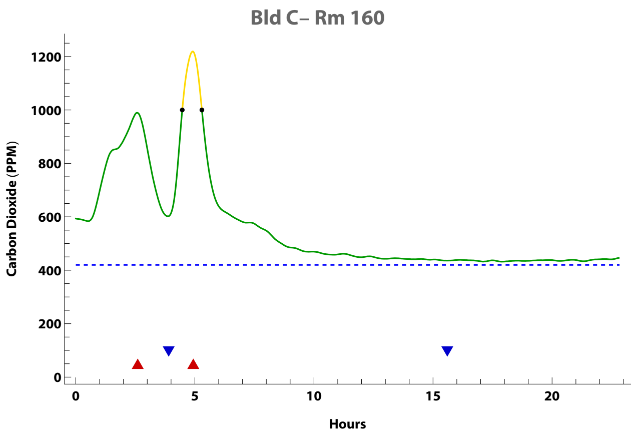

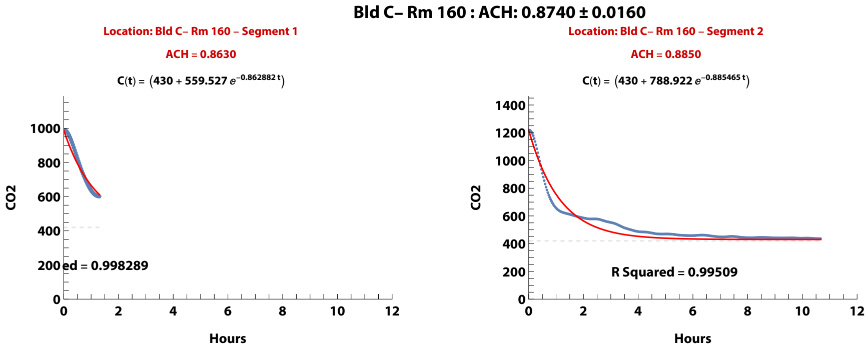

Zand, et al. (2023). Ventilation during COVID–19 in a school for students with intellectual and developmental disabilities (IDD) [Submitted]

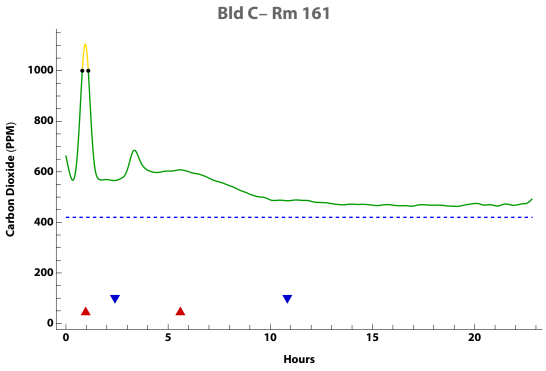

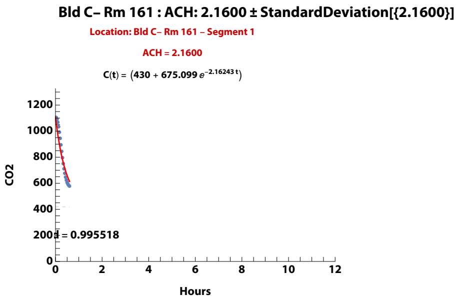

Zand, et al. (2023). Ventilation during COVID–19 in a school for students with intellectual and developmental disabilities (IDD) [Submitted]

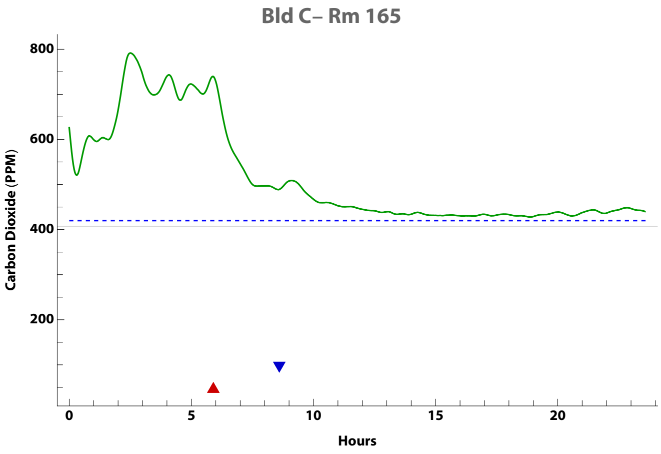

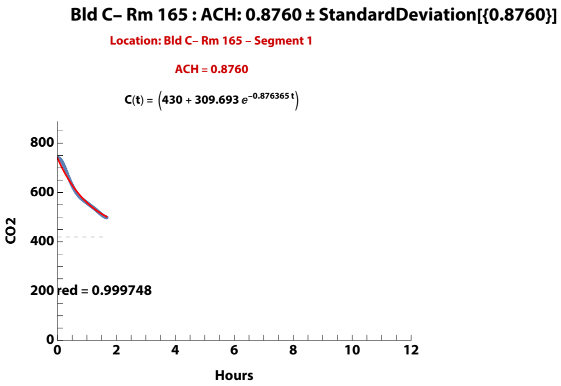

Zand, et al. (2023). Ventilation during COVID–19 in a school for students with intellectual and developmental disabilities (IDD) [Submitted]

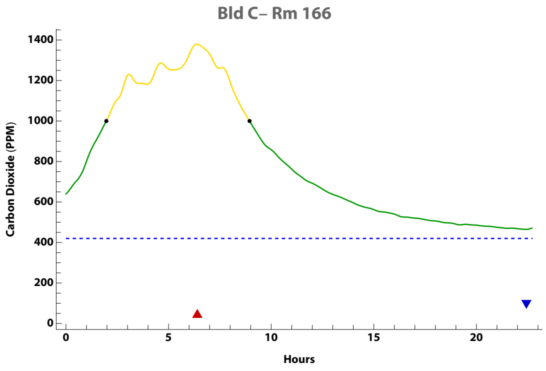

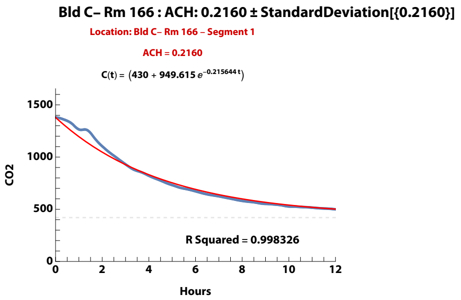

Zand, et al. (2023). Ventilation during COVID–19 in a school for students with intellectual and developmental disabilities (IDD) [Submitted]

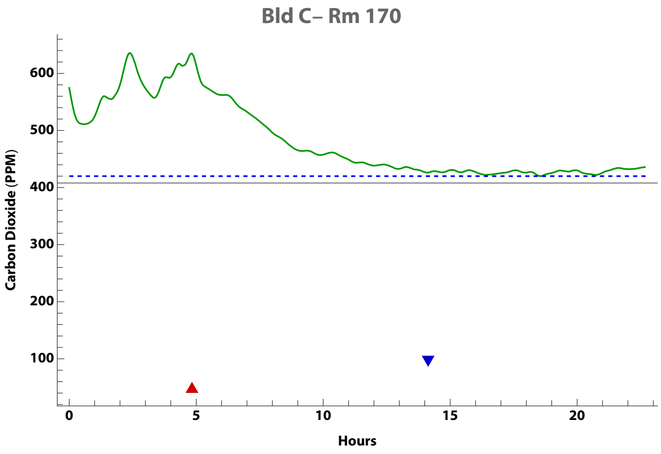

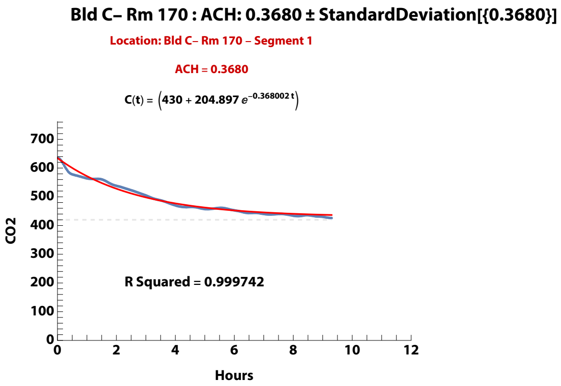

Zand, et al. (2023). Ventilation during COVID–19 in a school for students with intellectual and developmental disabilities (IDD) [Submitted]

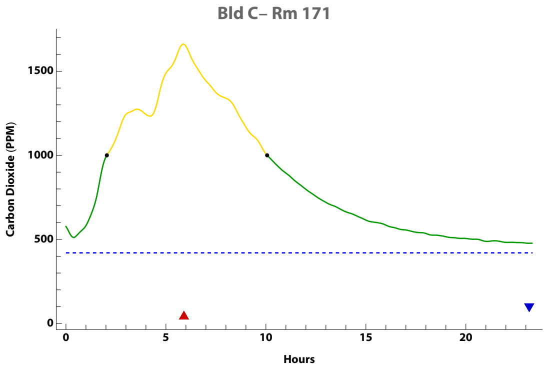

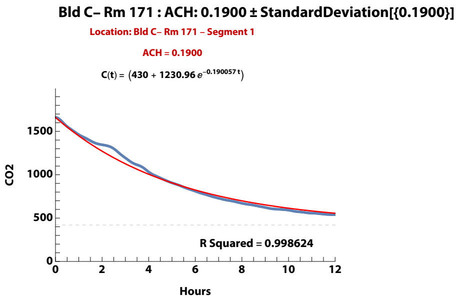

Zand, et al. (2023). Ventilation during COVID–19 in a school for students with intellectual and developmental disabilities (IDD) [Submitted]

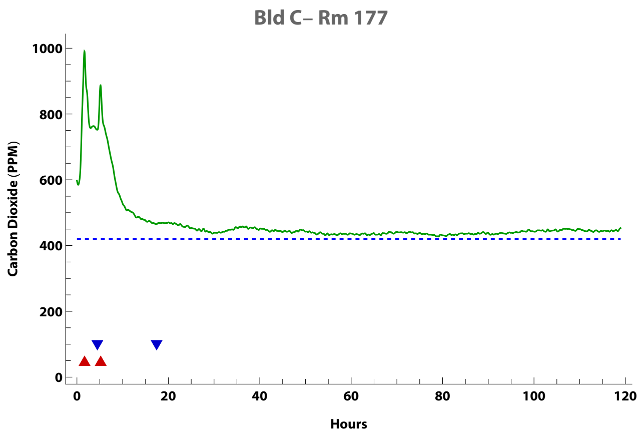

Bld C– Rm 177 : ACH: 0.3830 ± 0.1280

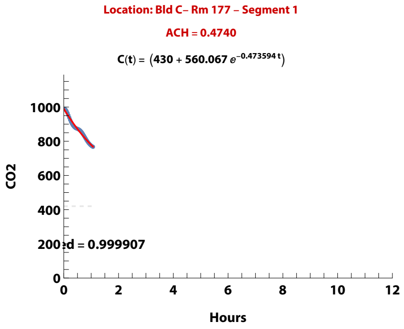

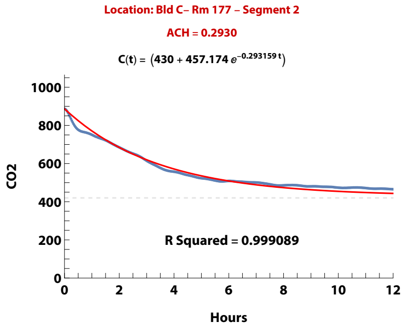

Zand, et al. (2023). Ventilation during COVID–19 in a school for students with intellectual and developmental disabilities (IDD) [Submitted]

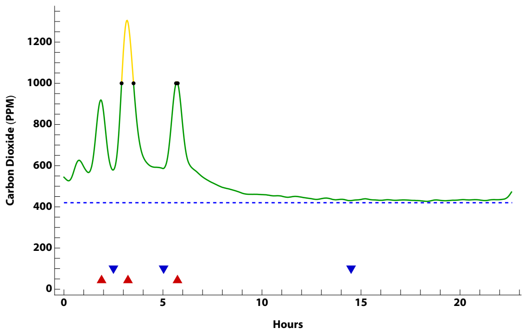

Bld C– Rm 184 : ACH: 1.6100 ± 0.2790

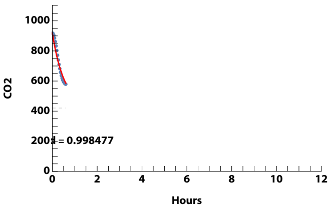

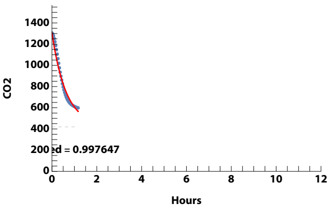

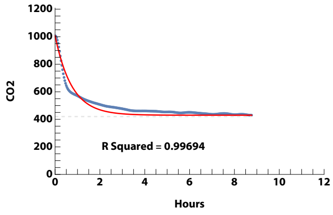

Zand, et al. (2023). Ventilation during COVID–19 in a school for students with intellectual and developmental disabilities (IDD) [Submitted]

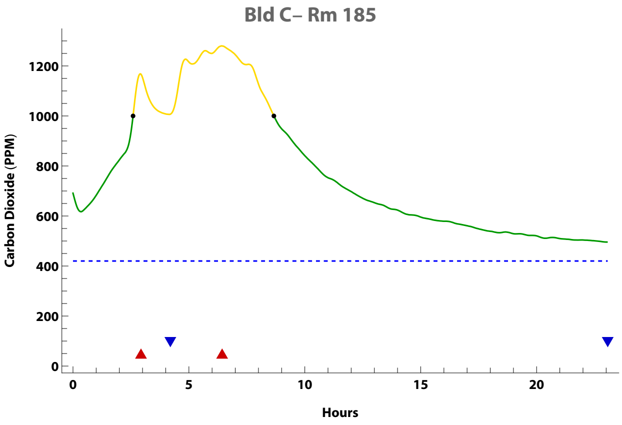

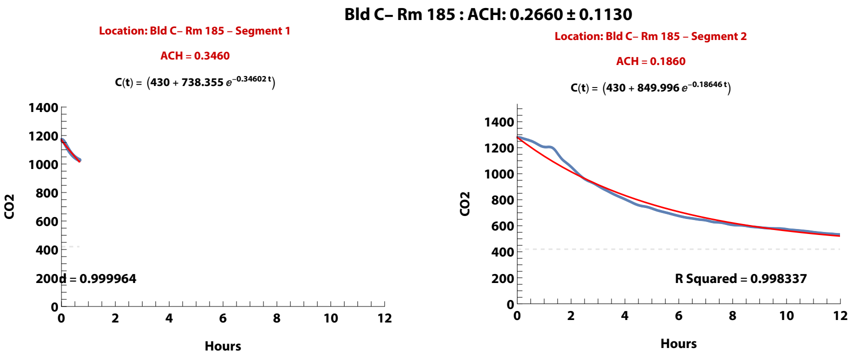

Zand, et al. (2023). Ventilation during COVID–19 in a school for students with intellectual and developmental disabilities (IDD) [Submitted]

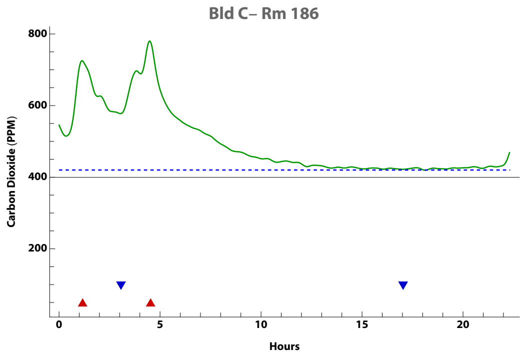

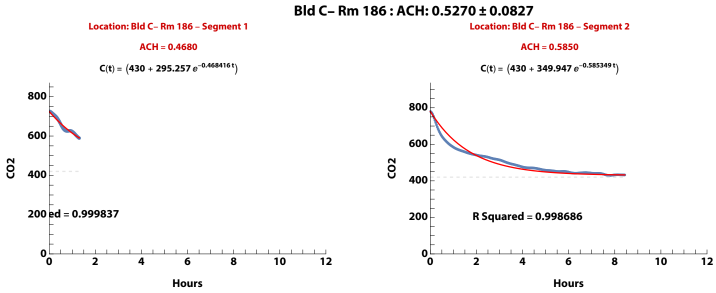

Zand, et al. (2023). Ventilation during COVID–19 in a school for students with intellectual and developmental disabilities (IDD) [Submitted]

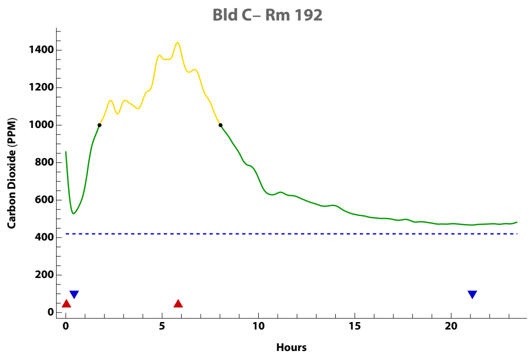

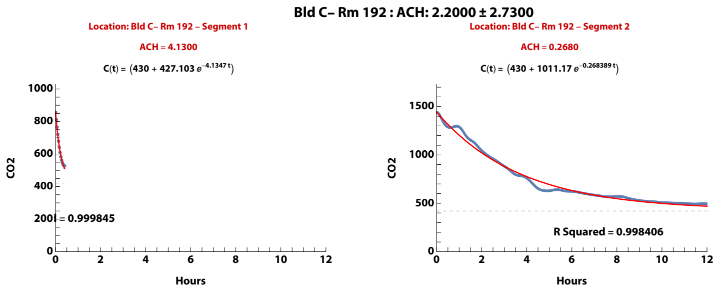

Zand, et al. (2023). Ventilation during COVID–19 in a school for students with intellectual and developmental disabilities (IDD) [Submitted]

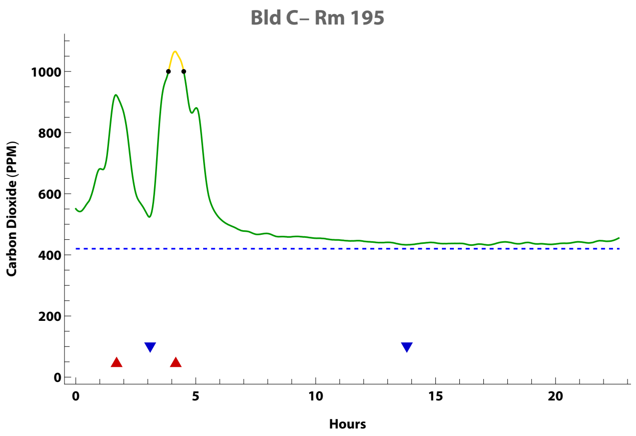

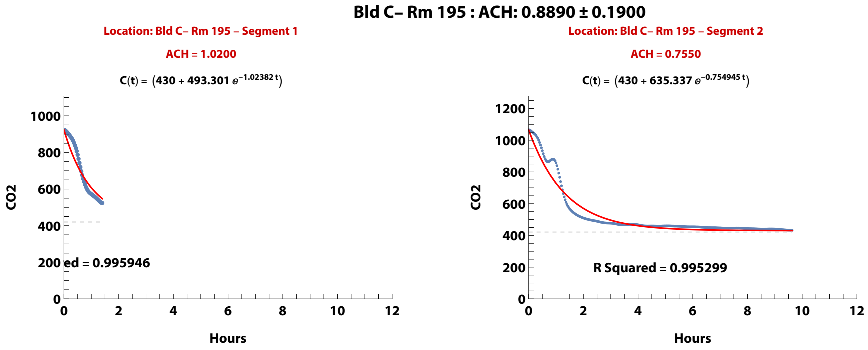

Zand, et al. (2023). Ventilation during COVID–19 in a school for students with intellectual and developmental disabilities (IDD) [Submitted]

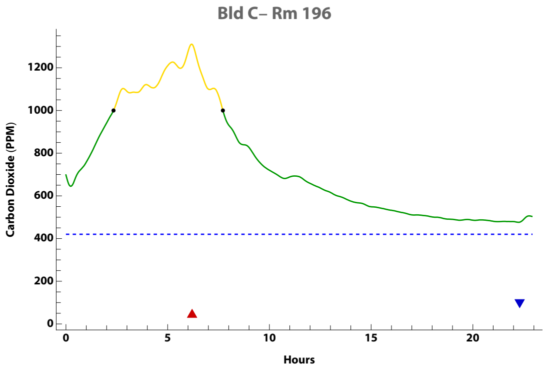

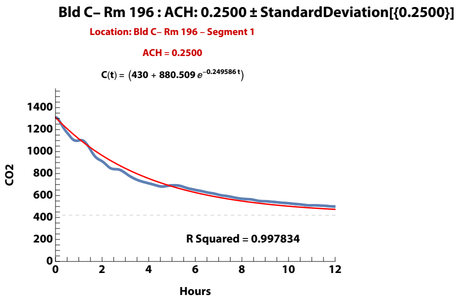

Zand, et al. (2023). Ventilation during COVID–19 in a school for students with intellectual and developmental disabilities (IDD) [Submitted]

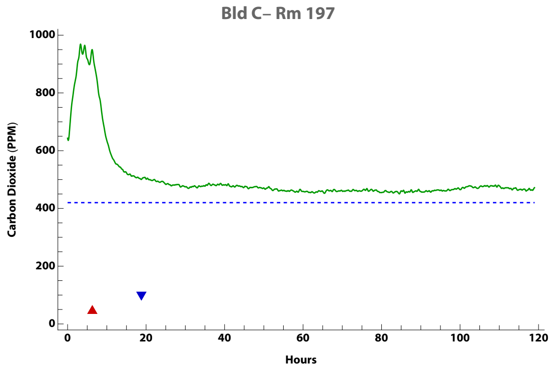

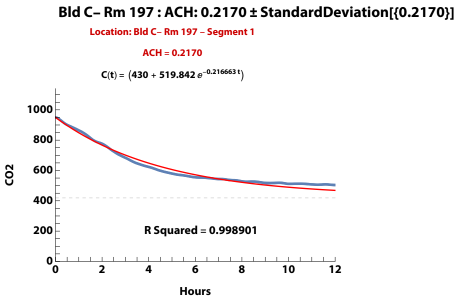

Zand, et al. (2023). Ventilation during COVID–19 in a school for students with intellectual and developmental disabilities (IDD) [Submitted]

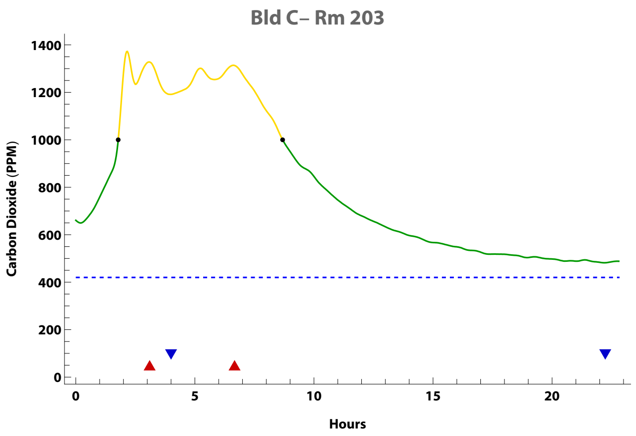

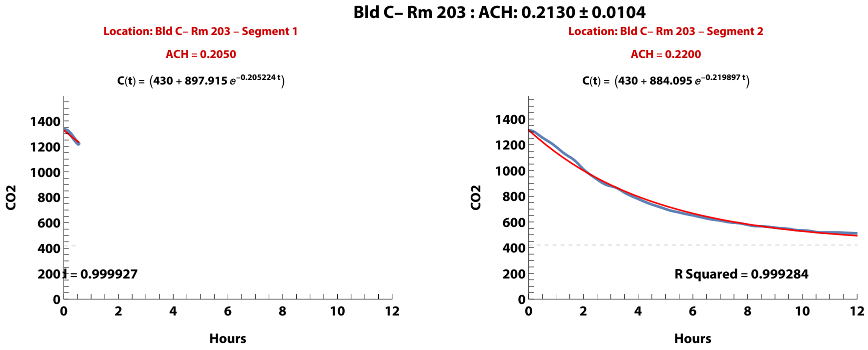

Zand, et al. (2023). Ventilation during COVID–19 in a school for students with intellectual and developmental disabilities (IDD) [Submitted]

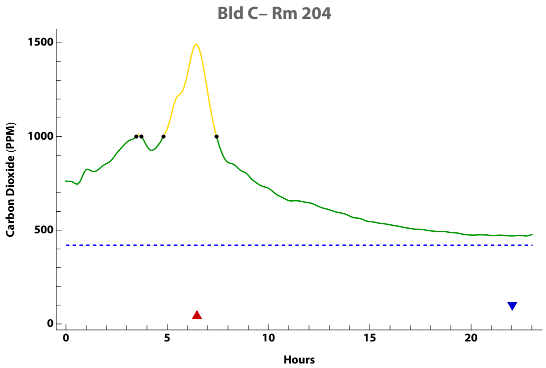

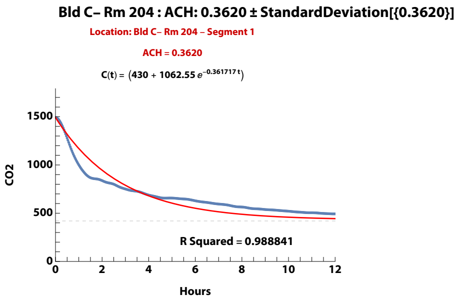

Zand, et al. (2023). Ventilation during COVID–19 in a school for students with intellectual and developmental disabilities (IDD) [Submitted]

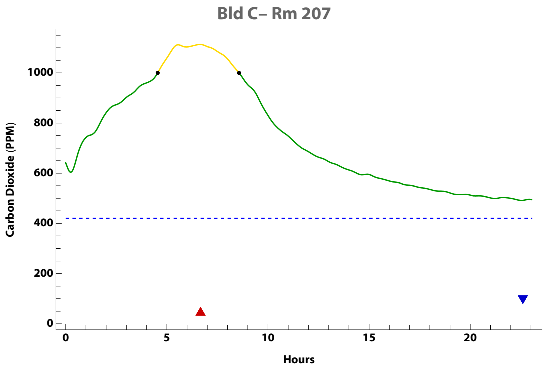

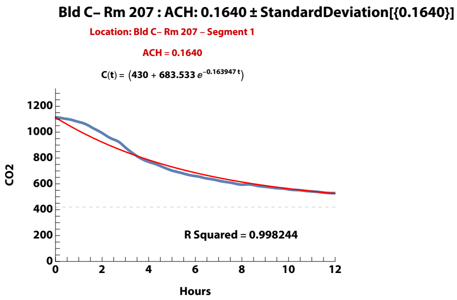

Zand, et al. (2023). Ventilation during COVID–19 in a school for students with intellectual and developmental disabilities (IDD) [Submitted]

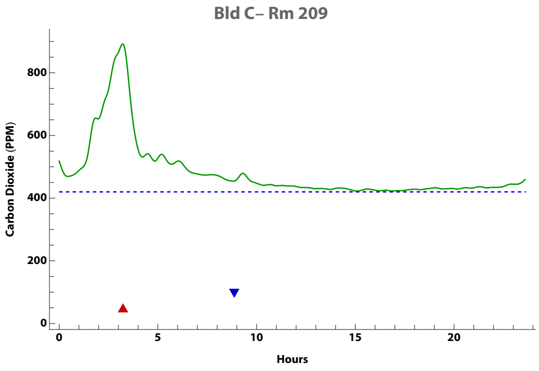

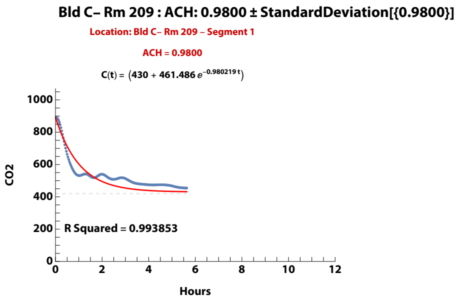

Zand, et al. (2023). Ventilation during COVID–19 in a school for students with intellectual and developmental disabilities (IDD) [Submitted]

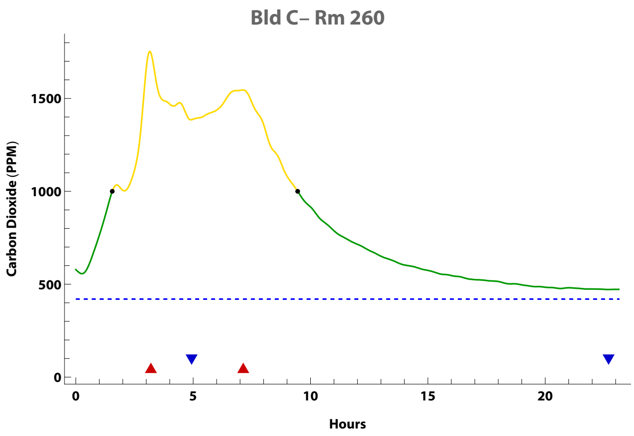

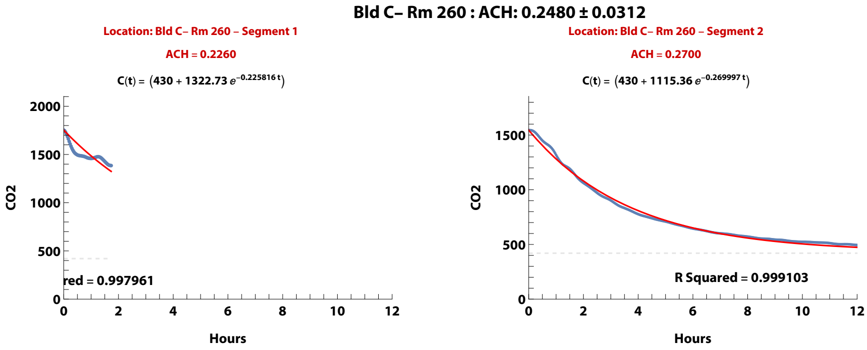

Zand, et al. (2023). Ventilation during COVID–19 in a school for students with intellectual and developmental disabilities (IDD) [Submitted]
